# Effectiveness of psychological interventions for parents of children eligible for paediatric palliative care: a systematic review and meta-analysis

**DOI:** 10.3389/fpsyg.2026.1775937

**Published:** 2026-03-03

**Authors:** Yolanda Álvarez-Pérez, Andrea Duarte-Díaz, Amado Rivero-Santana, Alejandra Abrante-Luis, Bernat Carreras, Diego Infante-Ventura, Vanesa Ramos-García, Alezandra Torres-Castaño, Estefanía Herrera-Ramos, Juan Luis Marrero Gómez, Lilisbeth Perestelo-Pérez

**Affiliations:** 1 Canary Islands Health Research Institute Foundation (FIISC), Tenerife, Spain; 2 Evaluation Unit (SESCS), Canary Islands Health Service (SCS), Tenerife, Spain; 3 Network for Research on Chronicity, Primary Care, and Health Promotion (RICAPPS), Tenerife, Spain; 4 The Spanish Network of Agencies for Health Technology Assessment and Services of the National Health System (RedETS), Tenerife, Spain; 5 Department of Adult Psychiatry and Psychology, Institute of Neurosciences (ICN), Hospital Clinic, Barcelona, Spain; 6 Paediatric Palliative Care Unit, Hospital Universitario Virgen del Rocío, Seville, Spain

**Keywords:** cancer, families, life-limiting, life-threatening illness, paediatric population, palliative care, parent, psychological interventions

## Abstract

**Background:**

Paediatric palliative care (PPC) is an early, holistic model addressing the physical, psychological, social, and spiritual needs of children with life-limiting or life-threatening conditions and their families. Parents frequently experience high psychological distress, and although psychological support is a core PPC component, empirical evidence on the effectiveness of interventions within this context remains limited, particularly across non-oncological conditions. This systematic review and meta-analysis aimed to evaluate the effectiveness of psychological interventions for parents of children eligible for PPC.

**Methods:**

A systematic search of MEDLINE, Embase, CINAHL, APA PsycINFO, and CENTRAL was conducted in July 2024 and updated in Embase in November 2025. Randomized controlled trials evaluating psychological interventions for parents of children under 18 years eligible for PPC were included. Methodological quality was assessed using the Cochrane Risk of Bias tool for randomized trials (RoB 2). The review was conducted in accordance with PRISMA guidelines, and random-effects meta-analyses were performed where appropriate.

**Results:**

Thirty-six studies were included. Although the review aimed to capture studies involving parents of children with various life-limiting conditions eligible for PPC, the evidence base was almost exclusively oncology-focused: only one study was conducted within a PPC context, and all studies involved parents of children with cancer. The psychological interventions were heterogeneous and frequently multimodal, with cognitive-behavioural therapy being the most common approach. Meta-analyses showed primarily short-term significant moderate-to-large effect sizes, indicating improvements in anxiety (*g* = −1.00; 95% CI: −1.49, −0.52; I^2^ = 94%), depression (g = −0.77; 95% CI: −1.15, −0.39; I^2^ = 85%), psychological distress (g = −0.39; 95% CI: −0.75, −0.04; I^2^ = 0%) and hope (*g* = 0.73; 95% CI: 0.20, 1.26; I^2^ = 0%). Evidence of long-term effects was limited, with improvements observed only for post-traumatic stress symptoms (*g* = −0.29; 95% CI: −0.55, −0.03; I^2^ = 0%).

**Discussion:**

The findings suggest that psychological interventions, particularly those based on cognitive behavioural approaches, are effective in reducing psychological distress and enhancing positive psychological outcomes in the short term among parents of children with cancer who are eligible for PPC. However, the scarcity of studies explicitly conducted within PPC settings highlights the need for further high-quality trials across a wider range of life-limiting and life-threatening conditions.

**Systematic review registration:**

https://www.crd.york.ac.uk/PROSPERO/view/CRD42024594171, CRD42024594171

## 1 Introduction

An estimated 21 million children worldwide, including newborns, infants, children, and adolescents up to 19 years of age, are thought to require a palliative care approach each year (Connor et al., 2017). Over the years, models of paediatric palliative care (PPC) have evolved towards a more integrated and holistic perspective. Both the World Health Organization and the American Academy of Pediatrics proposed comparable frameworks, emphasizing that PPC should be initiated early in the disease trajectory, regardless of whether curative treatment is being pursued (World Health Organization, 2018).

In this context, the Association for Children with Life-threatening or Terminal Conditions and their Families (ACT) and the Royal College of Paediatrics and Child Health (RCPCH) developed a framework to classify the diseases of children eligible for palliative treatment into four groups (Association for Children with Life-Threatening or Terminal Conditions and Their Families (ACT), 2009): (i) Group 1: Serious illnesses for which curative treatment exists but may fail (e.g., cancer; failure of vital organs such as the heart, liver, or lungs requiring transplantation); (ii) Group 2: Conditions requiring prolonged periods of intensive treatment aimed at managing the pathophysiology of the disease to sustain life, but where premature death remains possible (e.g., cystic fibrosis; Duchenne muscular dystrophy; epidermolysis bullosa); (iii) Group 3: Progressive diseases for which no curative treatment exists, only palliative options (e.g., neuromuscular or neurodegenerative disorders; progressive metabolic diseases; advanced cancer with metastasis at diagnosis); and (iv) Group 4: Irreversible but non-progressive conditions associated with severe disability, leading to extreme vulnerability to complications that may result in premature death (e.g., severe cerebral palsy; complex congenital malformation syndromes).

PPC represents a comprehensive form of healthcare that addresses not only the needs of children but also those of their caregivers, who are most often family members or other primary support figures. Parents of children with a serious illness often experience significant psychological distress, which can affect their own wellbeing and may interfere with their capacity to support and care for their child (Boyden et al., 2022). Qualitative research has shown that parents face constant anxiety about the possible loss of their child, live with uncertainty, must confront difficult decisions about treatment, and often manage feelings of guilt or helplessness (Verberne et al., 2019). Comprehensive care seeks to actively address the physical, psychological, social, and spiritual needs of both children and their families, as these dimensions have a direct impact on care quality, communication, and participation in decision-making (Verberne et al., 2019).

In line with this holistic perspective, a person-centred approach that includes the structured and formal inclusion of professionals specialized in clinical psychology is considered fundamental to PPC. Such integration ensures that care extends beyond physical and clinical indicators to encompass emotional and relational processes central to family wellbeing (Hord et al., 2014; Edlynn and Kaur, 2016; Benini et al., 2022; Thompson et al., 2024). Several European countries have incorporated psychological care into their PPC guidelines, regardless of the underlying diagnosis (National Guideline Alliance (UK), 2016; World Health Organization, 2018; Parlamento Europeo, 2022). However, although palliative care programs routinely acknowledge psychological and psychiatric needs, the systematic identification and continuous monitoring of emotional distress remain insufficient (Cress, 2024).

While many psychological interventions are designed to prevent or treat physical and mental health problems in the context of a long-term prognosis, the work of clinical psychologists and specialists in psycho-oncology and palliative care becomes equally important when recovery from illness is uncertain or unlikely (Abramson, 2022). Evidence indicates that psychological interventions in PPC aimed at alleviating emotional and behavioural symptoms are effective in reducing both physical and mental symptomatology (e.g., anxiety, depression, pain, fatigue). These interventions also contribute meaningfully to improving quality of life and well-being in families of children with serious illnesses (Law et al., 2019; Santini et al., 2022; Cardenas et al., 2023). Commonly used interventions include cognitive-behavioural therapy, mindfulness-based approaches, family therapy, acceptance and commitment therapy, dignity therapy, and grief and bereavement support, among others (Poltorak and Benore, 2006; Thompson and Kentor, 2021).

Despite the recognised importance of psychological care in addressing the complex needs of families affected by life-limiting conditions, clinical psychologists that working in paediatric palliative care contexts remain underrepresented in PPC service models, guidelines, and training programs (Thompson et al., 2023). This limited integration is reflected in the literature, where empirical studies evaluating psychological interventions specifically within PPC contexts are still scarce, despite their clear relevance and clinical necessity. Accordingly, this systematic review (SR) aims to evaluate the available scientific evidence on the effectiveness of psychological interventions, compared to usual care for parents of children eligible for PPC.

## 2 Materials and methods

The protocol of this SR was registered in the PROSPERO database (CRD42024594171). The review was conducted in accordance with the Preferred Reporting Items for Systematic Reviews and Meta-Analyses (PRISMA) guidelines (see Supplementary Table S1) (Page et al., 2021).

Given the limited number of studies evaluating psychological interventions delivered explicitly within formal PPC programmes, the scope of this SR was broadened to include studies involving parents of children with conditions that would make them potentially eligible for PPC, such as cancer, cystic fibrosis, or complex chronic illnesses. This decision was justified by the conceptual and clinical overlap in the psychological needs and care contexts of these groups. Studies on psychological interventions for children susceptible to PPC are addressed in another article.

### 2.1 Literature search

A systematic search of randomized controlled trials (RCTs) was conducted in the following electronic databases from inception date to July 2024: MEDLINE (Ovid), Embase (Elsevier), CINAHL (EBSCOhost), APA PsycINFO (EBSCOhost), and CENTRAL (Wiley). In addition, active alerts were maintained in Embase to identify new publications on the topic up to November 2025. The search filter developed by the Scottish Intercollegiate Guidelines Network to retrieve RCTs^1^ was applied in all databases except CENTRAL. Detailed search strategies are presented in Supplementary Table S2.

The search strategy was structured around two main concepts: (1) *paediatric palliative care*—defined as (palliative care OR terminal care OR hospice care OR end-of-life OR life-threatening OR long-term hospitalisation OR critically ill) AND (child OR infant OR teen OR adolescent OR prenatal OR newborn OR neonate OR medical complexity OR paediatric care OR paediatric complex care OR parent OR caregiver OR family)—and (2) *psychological interventions*—defined as (psychotherapy OR psychological intervention OR psychosocial intervention).

### 2.2 Eligibility criteria and study selection

Studies were eligible for inclusion if they met the following criteria: (1) RCTs; (2) studies involving primary caregivers (parents or legal guardians) of paediatric populations (under 18 years) potentially eligible for PPC (e.g., cancer, cystic fibrosis, Duchenne muscular dystrophy, complex chronic diseases); (3) studies evaluating the effectiveness of psychological interventions in which psychological outcomes (e.g., anxiety, depression, post-traumatic stress, psychological distress) or safety (e.g., adverse effects of the psychological interventions); (4) psychological interventions (e.g., cognitive-behavioural therapy, family therapy, acceptance and commitment therapy, dignity therapy), delivered individually or in groups, face-to-face or online, as standalone or part of multicomponent care, across inpatient, outpatient, or home care settings; (5) comparators such as usual care without psychological intervention; (6) for multicomponent interventions, only studies in which the isolated effect of the psychological component could be determined (e.g., component analyses or comparison arms without the psychological component) were included; and (7) articles published in English or Spanish.

Eligibility was defined broadly to include life-limiting or life-threatening conditions for which PPC may be indicated at any point across the disease trajectory. Given the heterogeneity of PPC populations, no restrictions were applied regarding specific diagnoses at the search or study selection stages.

The exclusion criteria were: (1) studies involving only paediatric populations (this data is presented in another publication); (2) involving family members who are not the parents or primary caregivers (e.g., siblings); (3) parents or primary caregivers of paediatric populations in acute care settings (e.g., emergency departments or intensive care units), of paediatric cancer survivors or of deceased children; and (4) healthcare or clinical professionals.

Study selection was performed independently by two reviewers, with any disagreements resolved through discussion involving a third reviewer. Initially, titles and abstracts were screened to assess their relevance to the inclusion criteria. Full texts of potentially eligible articles were then examined, and studies meeting all inclusion criteria were selected. Additionally, reference lists of the included studies were reviewed to identify further relevant RCTs.

### 2.3 Data extraction and assessment of methodological quality

Data extraction was carried out in an Excel file including information from each study (e.g., dates of data collection), sample characteristics (e.g., age, gender, child diagnosis), study setting (e.g., PPC unit, hospital, home), study design (e.g., comparison condition), intervention details (e.g., number sessions, format, duration, provider), and intervention components. Methodological quality was assessed using the Cochrane risk-of-bias tool for randomized trials (RoB 2) (Sterne et al., 2019). Data extraction and risk-of-bias were conducted independently by two reviewers. A third review author was consulted regarding any discrepancies, and these were resolved by discussion until consensus was reached.

### 2.4 Analysis

A meta-analysis (MA) was conducted for each outcome whenever data were available from at least two studies for the post-treatment measure, using Review Manager (RevMan, version 5.4.1). MA were conducted using a random-effects model (DerSimonian and Laird method) as substantial clinical and methodological heterogeneity across studies was anticipated.

For all outcomes, post-intervention or follow-up scores were used. When outcome measures differed in their scoring direction (i.e., higher scores indicating better versus worse outcomes), the sign of the effect size was reversed for scales in which higher scores represented better outcomes, so that negative standardized mean differences consistently reflected symptom reduction or improvement across all analyses. Statistical heterogeneity between studies included in the MA was assessed using the Higgins I^2^ value (Higgins and Thompson, 2002). Subgroup analyses by age group, type or severity of the children’s illness (e.g., different types of cancer), or type of intervention received (e.g., type, number and duration of sessions, frequency) could not be performed due to the limited number of studies available for each outcome. For some outcome measures, it was only possible to perform subgroup analyses based on the assessment instrument used and the type of population evaluated (both parents versus mothers only).

Publication bias was visually analysed using the funnel plot and statistically with the Egger’s test (Sterne et al., 2001), when there were eight or more studies. The results of studies that could not be included in the MA, as well as outcomes reported in only one study, are presented narratively.

## 3 Results

### 3.1 Results of the search

**Figure 1:**
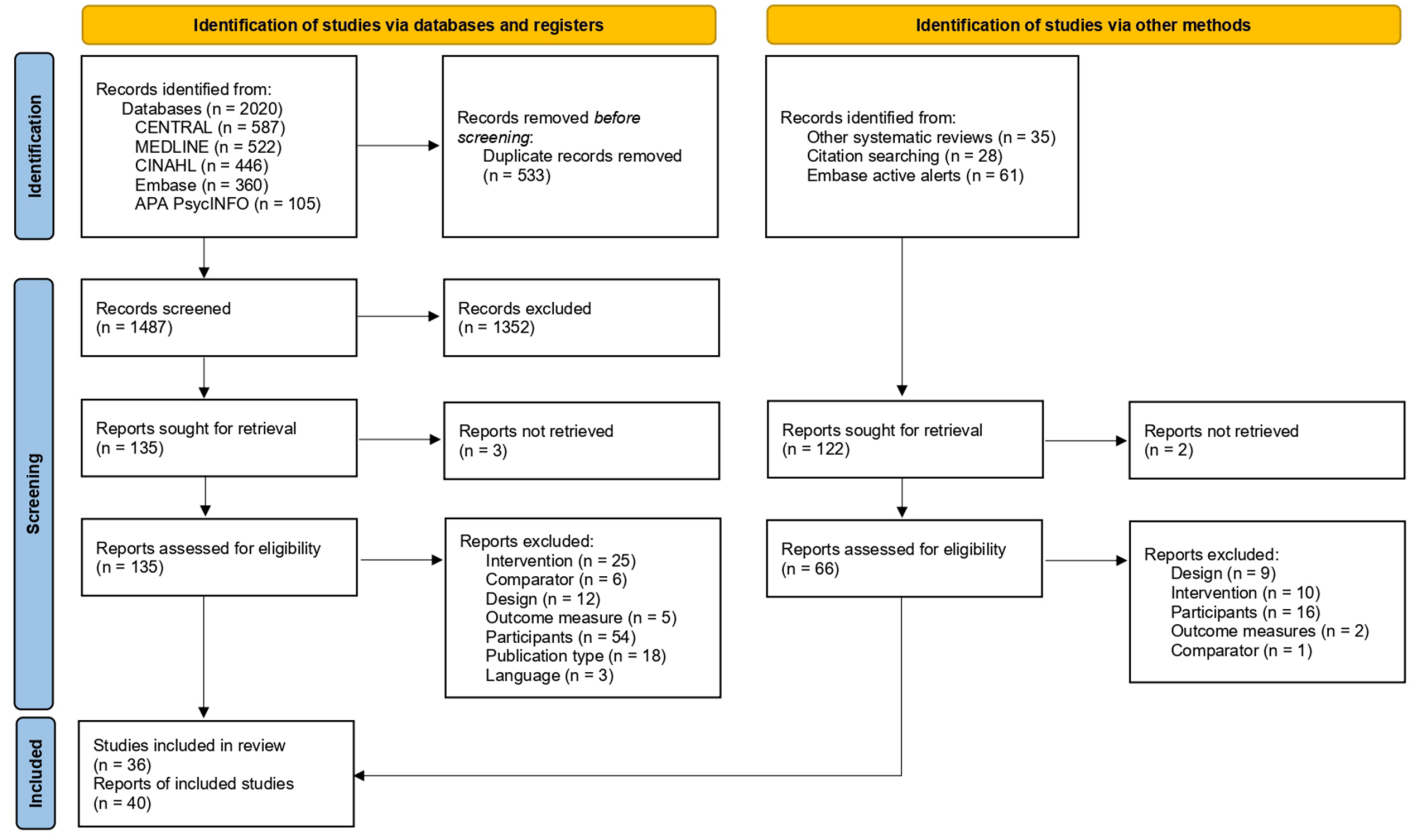
Flowchart of study selection according to PRISMA guidelines.

The initial search of RCTs in the electronic databases yielded 1,487 references without duplicates. After screening by title and abstract, 135 full-text articles were assessed for eligibility. Twenty-eight additional records were identified from other systematic reviews, citation searching, and Embase active alerts. Forty references, corresponding to 36 studies involving parents of children with cancer, were finally included (Figure 1).

### 3.2 Methodological quality

According to the Cochrane Collaboration’s tool, a high risk of bias in any of the assessed domains, or an unclear risk of bias in multiple domains, led to an overall rating of high risk of bias (Sterne et al., 2019). Consequently, none of the studies included were rated as having a low risk of bias (see Supplementary Figure S1). Several studies did not provide information on the blinding procedures carried out, but given the characteristics of the interventions, the intervention provider could not be blinded to the conditions of the groups. Most studies used patient-reported measures (e.g., quality of life, anxiety, depression), and the evaluator was not blinding.

### 3.3 Study characteristics

The main characteristics of the included studies are summarised in Table 1. Of the 36 studies included, two (in four references) (Ebrahimi et al., 2019; Cho et al., 2023; Akard et al., 2021a,b) evaluated the effectiveness of psychological interventions in both children and their parents. Although the review was designed to capture studies involving parents of children eligible for PPC across a range of life-limiting conditions, all included RCT were conducted in paediatric oncology settings. Only one study, reported across multiple publications (Cho et al., 2023; Akard et al., 2021a,b) was explicitly conducted in the context of PPC with children with advanced cancer and their parents. Sixteen studies (44.4%) involved only mothers.

**Table 1:** Summary of main characteristics of the included studies.

Study (year) country	Setting	Participants		Intervention				Control	Duration	Follow up	Outcomes (scale)
		Characteristics of Participants	Children eligible for PPC	Content	Type (format)	Number of sessions (frequency) and duration (min)	Provider				
Bahrami et al. (2020), Iran	Hospital	Parents of children with cancer *N* = 60 (GI: 30; GC: 30) Females: N.R. Mean age (years) ± SD: 32.38 ± 5.0 Age range: N.R.	*N* = 60 (GI: 30; GC: 30) Females: 28.33% Mean age (years) ± SD: 7.75 ± 3.6 Type of cancer: leukaemia (65%), Wilms tumour (10%), lymphoma (6.67%), brain tumour (6.67%), neuroblastoma (6.67%), sarcoma (5%)	Emotional regulation training based on Gross’s psychological model	N.R. (face-to-face)	8 sessions (1 per week) 90 min/session	Psychiatric nurse and researcher	Usual care (unspecified)	4 weeks	3 months	Anxiety (BAI)
Cernvall et al. (2015, 2017), Sweden	Hospital	Parents of children with cancer *N* = 58 (GI:31; GC:27) Females: 67% Mean age (years) ± SD: 38 ± 7.2 Age range: N.R.	*N* = 46 (GI: 25; GC: 21) Females: 54% Mean age (years) ± SD: 5 ± 9.0 Type of cancer: leukaemia (52%), sarcoma (17%), central nervous system tumours (15%), lymphoma (7%), other malignant tumours (9%)	Online guided self-help program, based on the Cognitive-behavioural therapy + Usual care	Individual (online-Website)	9 sessions (modules) (1 per week) N.R. min/session	One psychologist and two non-licensed psychologists with master’s degrees, supervised by the first	Usual care (standard psychosocial services)	10 weeks	12 months	Post-traumatic stress (PCL) Depression (BDI) Anxiety (BAI)
Damreihani et al. (2018), Iran	Hospital	Mothers of children with cancer *N* = 50 (GI: 25; GC: 25) Females: 100% Mean age (years) ± SD: 35.55 ± 5.6 Age range: N.R.	*N* = N.R. Females: N.R. Mean age (years) ± SD: N.R. Type of cancer: leukaemia (100%)	Positive psychology intervention (task-oriented to improve skills and positive emotions)	Group (face-to-face)	6 sessions (N.R. frequency) 120 min/session	N.R.	No intervention	N.R.	1 month	Hope (SHS) Psychological Well-being (PWBS) Purpose in Life (MLQ) Life Satisfaction (SWLS)
Delavari et al. (2014), Iran	Hospital	Mothers of children with cancer *N* = 30 (GI: 15; GC: 15) Females: 100% Mean age (years) ± SD: N.R. Age range: N.R.	*N* = N.R. Females: N.R. Mean age (years) ± SD: N.R. Type of cancer: N.R.	Logotherapy	Group (face-to-face)	9 sessions (1 per week) 90 min/session	Therapist (unspecified)	No intervention	9 weeks	–	Depression (BDI) Anxiety (BAI)
Gholamian et al. (2021), Iran	Hospital	Mothers of children with cancer *N* = 44 (GI: 22; GC: 22) Females: 100% Mean age (years) ± SD: 37.33 ± 6.58 Age range: N.R.	N = 42 (GI: 20; GC: 22) Females: N.R. Mean age (years) ± SD: N.R. Type of cancer: leukaemia (50%), haemolytic anaemia (17%), brain tumour (14%), sarcoma (12%), neck tumour (7%)	Peer-to-peer self-help group program (psychoeducation, thought re-evaluation, anxiety management, etc.)	Group (face-to-face)	8 sessions (N.R. frequency) 45–60 min/session + follow-up calls 20–40 min each	Psychiatrist and oncology nurse	Usual care (cancer information leaflet)	12 weeks	–	Anxiety (HAM-A)
Hoekstra-Weebers et al. (1998), Netherlands	Hospital	Parents of children with cancer N = 81 (GI: 39; GC: 42) Females: 50.62% Mean age (years) ± SD: 36.6 ± 5.4 Age range: 24–53 years	*N* = 41 (N.R. per group) Females: 43.9% Mean age (years) ± SD: 6.4 4.7 Age range: 0–16 years Type of cancer: leukaemia (42%), lymphoma (17%), tumour de Wilms (11%), brain tumour (10%), sarcoma (10%), endocrine tumour (5%), other types of cancer (5%)	Guided intervention focused on cognitive-behavioural techniques and psychoeducation (problem solving, assertiveness, etc.) + Usual care	Individual (face-to-face)	8 sessions (1 every 3 weeks) 90 min/session	Psychologist	Usual care (routine medical care was provided by paediatricians and nurses. A social worker offered psychosocial support after diagnosis, and subsequent contact was initiated by the family)	24 weeks	6 months	Anxiety (STAI-S) Psychiatric symptoms (SCL-90) General Health Questionnaire (GHQ)
Hoseinzadeh et al. (2019), Iran	Hospital	Mothers of children with cancer N = 48 (GI: 24; GC: 24) Females: 100% Mean age (years) ± SD: 34.26 ± 6.77 Age range: N.R.	*N* = N.R. Females: N.R. Mean age (years) ± SD: N.R. Type of cancer: N.R.	Resilience-based intervention (self-esteem, problem-solving, emotional management, etc.)	Group (face-to-face)	6 sessions (N.R. frequency) 60–90 min/session	N.R.	No intervention	N.R.	3 months	Coping (CHIP)
Jamali et al. (2019), Iran	Hospital	Mothers of children with cancer *N* = 74 (GI: 36; GC: 38) Females: 100% Mean age (years) ± SD: N.R. Age range: N.R.	*N* = N.R. Females: N.R. Mean age (years) ± SD: N.R. Type of cancer: leukaemia (100%)	Peer-led psychoeducation group program focused on resilience (goal setting, stress management, resilience-enhancing strategies, etc.)	Group (face-to-face)	5 sessions (1 daily for 5 days) 90 min/session	Peer educator: mother of a minor who had already finished chemotherapy, trained in group sessions (3 sessions of 1 h each)	Usual care (unspecified)	5 días	2 months	Resilience (CD-RISC)
Jay and Elliott (1990), USA	Hospital	Parents of children with cancer undergoing bone marrow biopsy or lumbar punctures *N* = 72 (GI: 24; GC: 24) Females: 79% Mean age (years) ± SD: N.R. Age range: 22–62 years	*N* = N.R. Females: N.R. Mean age (years) ± SD: N.R. Type of cancer: leukaemia (100%)	Stress inoculation for parents (modelling, self-instruction, relaxation and guided imagery)	Individual (face-to-face)	1 session 45 min	PhD students in psychology	No intervention	N.A.	–	Distress (PBS) Anxiety (STAI-S)
Jin et al. (2023), China	Hospital and Home	Parents of children with cancer *N* = 40 (GI: 20; GC: 20) Females: 80% Mean age (years) ± SD: 34.70 ± 4.53 Age range: N.R.	N = 40 (GI: 20; GC: 20) Females: 45% Mean age (years) ± SD: 7.35 ± 3.74 Type of cancer: haematological (82.5%), solid tumours (17.5%)	Acceptance and Commitment Therapy + Usual care	Individual (face-to-face or online)	4 sessions (1 per week) 45–60 min/session	PhD student in nursing with training in acceptance and commitment therapy (14-day course)	Usual care (unspecified)	4 weeks	–	Stress (DASS-21) Quality of life (PedsQL-Parent Scale) Emotional impact of the child’s illness (PECI)
Joosten et al. (2024), Netherlands	Hospital and Home	Parents of children with cancer *N* = 89 (GI: 43; GC: 46) Females: 86% Mean age (years) ± SD: 41.9 ± 7.45 Age range: N.R.	N = 89 (GI: 43; GC: 46) Females: N.R. Mean age (years) ± SD: 10.1 ± 5.65 Type of cancer: haematological (48.3%), solid tumours (29.2%), brain tumour (22.5%)	Active coping skills program (Op Koers Online) based on cognitive-behavioural and acceptance and commitment techniques	Group (face-to-face or online)	6 sessions (1 per week) 90 min/session + 1 booster session 5 months after the 1st session	Social worker or psychologist, supervised by a health psychologist	No intervention	6 weeks	6 months	Anxiety y Depression (PROMIS) Distress (DT-P) Emotional reactions related to the illness (SSER-Q) Coping (OK-Q y CCSS-P)
Kaboudi et al. (2018), Iran	Hospital	Mothers of children with cancer *N* = 60 (GI: 30; GC: 30) Females: 100% Mean age (years) ± SD: 38.85 ± 11.89 Age range: N.R.	*N* = N.R. Females: N.R. Mean age (years) ± SD: N.R. Type of cancer: leukaemia (100%)	Resilience training program (familiarization with the concept of resilience and the characteristics of resilient people, internal and external support factors, and ways to build resilience)	Group (face-to-face)	9 sessions (1 per week) 60 min/session	Doctor of Psychology with specific training in the resilience program	No intervention	9 weeks	–	Coping (SCQ) Stress (PSS)
Kazak et al. (2005), USA	Hospital	Parents of children with cancer *N* = 38 (GI: 18; GC: 20) Females: 52.6% Median age: 37 years	*N* = N.R. Females: N.R. Mean age (years) ± SD: N.R. Type of cancer: leukaemia (42%), solid tumours (31.6%), brain tumours (26.4%)	Adapted cognitive-behavioural therapy and family therapy program + Usual care	Individual (face-to-face)	3 sessions (depending on family availability) 45 min/session	Psychologists with specific training in the program	Usual care (routine psychosocial care that included the assignment of a social worker, who provided resources and information about diagnosis and treatment and offered support. Psychologists were available by referral to address behavioural concerns of the child and family)	N.R.	–	Anxiety (STAI-S)
Khosrobeigi et al. (2022), Iran	Hospital	Parents of children with cancer *N* = 30 (GI: 15; GC: 15) Females: N.R. Mean age (years) ± SD: 41.49 ± 4.11 Age range: N.R.	*N* = N.R. Females: N.R. Mean age (years) ± SD: N.R. Type of cancer: N.R.	Compassion Therapy (mindfulness, compassionate thinking and behaviour, breathing, mental visualization, empty chair technique, etc.)	Individual (face-to-face)	8 sessions (1 per week) 90 min/session	Psychotherapist with experience and training in the field	No intervention	8 weeks	–	Resilience (CD-RISC) Hopelessness (BHS)
Latifi et al. (2021), Iran	Hospital	Mothers of children with cancer *N* = 100 (GI: 50; GC: 50) Females: 100% Mean age (years) ± SD: 34.33 ± 6.42 Age range: N.R.	*N* = 100 (GI: 50; GC: 50) Females: 44% Mean age (years) ± SD: 5.43 ± 2.89 Type of cancer: N.R.	Cognitive-emotional intervention	Group (face-to-face)	5 sessions (2 per week) 60–90 min/session	Nurse with a master’s degree in psychiatric nursing and clinical experience in oncology, under the supervision of a person with a doctorate in counselling	Usual care (unspecified)	2–3 weeks	4 months	Distress (KPD)
Luo et al. (2021a), China	Hospital and Home	Parents of children with cancer *N* = 103 (GI: 52; GC: 51) Females: 69.9% Mean age (years) ± SD: 33.6 ± 5.2 Age range: N.R.	*N* = 89 (GI: 43; GC: 46) Females: N.R. Mean age (years) ± SD: 5.9 ± 3.9 Type of cancer: haematological (67%), solid tumours (33%)	Online resilience training program with content on relaxation, meditation, mindful breathing, problem-solving, cognitive strategies for thought management, etc.	Individual (mobile App)	8 online sessions/publications (1 per week) + 15 min/session self-administered (online)	Self-administered (online) Content developed by: professors of psychology, psycho-oncology, and paediatric oncology; paediatric oncology nurses; and a researcher trained in resilience programs	Usual care (health education in online format: information for the care of children with cancer: oral care, symptom management, infection prevention, etc.)	8 weeks	6 months	Resilience (CD-RISC) Depression (SRDS) Quality of life (SF-6D)
Manne et al. (2016), USA	Hospital	Parents of children with cancer undergoing hematopoietic stem cell transplantation *N* = 218 (GI: 110; GC: 108) Females: 88% Mean age (years) ± SD: 37.4 ± 8.1 Age range: N.R.	*N* = 89 (GI: 43; GC: 46) Females: N.R. Mean age (years) ± DE: 5.9 ± 3.9 Type of cancer: haematological (67%), solid tumours (33%)	Brief cognitive-behavioural therapy (relaxation, problem-solving, cognitive restructuring, etc.)	Individual (face-to-face)	5 sessions (N.R. frequency) 60 min/session	Psychologists	Usual care (materials on the emotional responses of caregivers and children to bone marrow transplantation, issues related to hospitalisation and communication with the child)	2–3 weeks	6 and 12 months	Depression (BDI) Anxiety (BAI) Post-traumatic stress (IES) Psychological well-being (MHI)
Marsland et al. (2013), USA	Hospital and Home	Parents of children with cancer *N* = 45 (GI:30; GC: 15) Females: 98% Mean age (years) ± SD: 41 ± 8 Age range: 28–53 years	*N* = 45 (GI: 30; GC: 15) Females: N.R. Mean age (years) ± SD: 13.05 ± 3 Type of cancer: leukaemia o lymphoma (78.4%), solid tumours (21.6%)	Multimodal intervention for stress management based on cognitive-behavioural techniques (progressive muscle relaxation, deep breathing, emotional stress management, visual imagery, etc.)	Individual (face-to-face)	6 sessions (1 every 2–3 weeks) 30–60 min/session	Physicians	Usual care (access to a social worker if required, but not preventive psychosocial intervention)	17–26 weeks	–	Depression (BDI) Anxiety (STAI-S) Stress (PSS) Post-traumatic stress (IES)
Marsland et al. (2020), USA	Hospital and Home	Mothers of children with cancer *N* = 120 (GI: 60; GC: 60) Females: 100% Mean age (years) ± SD: 36 ± 8 Age range: 19–57 years	*N* = 120 (GI: 60; GC: 60) Females: N.R. Mean age (years) ± SD: N.R. Type of cancer: leukaemia (55%), sarcoma (20.8%), neuroblastoma (9.2%), lymphoma (7.5%), other types of cancer (7.5%)	Multimodal intervention for stress management based on cognitive-behavioural techniques (progressive muscle relaxation, deep breathing, emotional stress management, visual imagery, etc.)	Individual (face-to-face + phone calls)	12 sessions (6 in-person sessions of 50–60 min each + 6 phone calls of 30 min each)	Physicians and psychologists	Usual care (access to a social worker if required, but not preventive psychosocial intervention)	8–30 weeks	12 months	Depression (BDI) Anxiety (STAI-S) Stress (PSS) Post-traumatic stress (IES) Quality of life (RAND-36)
Mullins et al. (2012), USA	Hospital	Mothers of children with cancer *N* = 52 (GI: 27; GC:25) Females: 100% Mean age (years) ± SD: 35.09 ± 6.93 Age range: 22–55 years	*N* = 52 (GI: 76; GC: 25) Females: 46.2% Mean age (years) ± SD: 8.19 ± 4.54 Type of cancer: N.R.	Cognitive-behavioural therapy (cognitive coping, problem-solving, etc.)	Individual (face-to-face)	12 sessions (1 per week) 45–60 min/session	Psychologist + nurse	Usual care (standard psychosocial care) (*ad hoc* care from the oncology team, including a psychologist, if needed during hospitalisation and outpatient visits)	12 weeks	3 months	Post-traumatic stress (IES) Psychiatric symptoms (SCL-90) Caregiver burden (CMCC)
Ozturk and Katikol (2024), Türkiye	Hospital and Home	Mothers of children with cancer *N* = 50 (GI: 25; GC: 25) Females: 100% Mean age (years) ± SD: 36.88 ± 5.4 Age range: 23–47 years	*N* = 50 (GI: 25; GC: 25) Females: 54% Mean age (years) ± SD: 11.72 ± 3.61 Type of cancer: lymphoma (38%), leukaemia (36%), sarcoma de Ewing (14%), osteosarcoma (10%), ependymoma (2%)	Online relaxation program based on cognitive-behavioural techniques and mindfulness (progressive muscle relaxation, guided imagery, mindfulness-based cognitive coping techniques, etc.)	Individual (mobile App) + Group (face-to-face)	7 sessions: 2 sessions face-to-face (1 per week) N.R. min/session + 5 at home sessions (practice at least twice a week)	Online program (mHealth) unspecified	Usual care (routine psychosocial support)	8 weeks	–	Anxiety (STAI-S) Coping (SCS)
Park et al. (2023), South Korea	Hospital and Home	Parents of children with cancer *N* = 40 (GI: 20; GC: 21) Females: 100% Mean age (years) ± SD: 42.25 ± 4.5 Age range: N.R.	*N* = 40 (GI: 20; GC: 21) Females: 46% Mean age (years) ± SD: 10.42 ± 4.4 Type of cancer: leukaemia (80.49%), lymphoma (19.51%)	Online program for promoting family resilience (emotional expression, coping strategies, etc.)	Individual (online)	4 sessions (1 per week) 70 min/session	Paediatric Nurse	Usual care (unspecified)	4 weeks	1 month	Resilience (WQ) Depression (BDI)
Phiri et al. (2025), China	Hospital	Parents of children with cancer *N* = 118 (GI: 59; GC: 59) Females: 84.7% Mean age (years) ± SD: 34.25 ± 9.03	*N* = 118 (GI: 59; GC: 59) Females: 38.1% Mean age (years) ± SD: 7.95 ± 4.18 Type of cancer: leukaemia (35.6%), lymphoma (41.5%), retinoblastoma (0.8%), Wilms tumour (18.6%), other types of cancer (3.4%)	Multicomponent group psychoeducational intervention (health education, coping skills training, stress management techniques training and psychological support)	Group (face-to-face)	36 sessions (6 per week) 60 min/session	Nurse	Usual care (routine health education, material support, food, and transport during follow-up visits)	6 weeks	3 months	Anxiety (GAD-7) Depression (PHQ-7) Quality of life (SF-12) Coping (COPE)
Porter et al. (2019), USA	Hospital	Parents of children with cancer *N* = 42 (GI:30; GC: 12) Females: 50% Mean age (years) ± SD: 39 ± 7.05 Age range: N.R.	*N* = 120 (GI: 60; GC: 60) Females: 45% Mean age (years) ± SD: 8.1 ± 5.3 Type of cancer: leukaemia (38%), central nervous system tumours (24%), sarcoma (14%), other types of cancer (24%)	Couples therapy (problem-solving, emotional management, etc.)	In pairs (face-to-face)	6 sessions (1 every 2 weeks) 60 min/session	Social Worker	Active control (health education: importance of rest, nutrition, physical activity, etc. Six sessions of 60 min each.)	12 weeks	–	Resilience (PTGI) Emotional impact of the child’s illness (PECI)
Pouraboli et al. (2019), Iran	Hospital	Parents of children with cancer *N* = 120 (GI: 60; GC: 60) Females: N.R. Mean age (years) ± SD: 37.48 ± 11.76 Age range: N.R.	*N* = N.R. Females: N.R. Mean age (years) ± SD: N.R. Type of cancer: leukaemia (100%)	Progressive Muscle Relaxation Training	Group (face-to-face)	8 sessions (2 per week) 20 min/session	Researcher	No intervention	4 weeks	–	Anxiety (STAI-S) Fatigue (BFI) Sleep quality (PSQI)
Safarabadi-Farahani et al. (2016), Iran	Hospital	Parents of children with cancer N = 62 (GI: 31; GC: 31) Females: 95% Mean age (years) ± SD: 33.73 ± 6.27 Age range: 24–47 years	*N* = 62 (GI: 31; GC: 31) Females: N.R. Mean age (years) ± SD: 6.04 ± 4.07 Type of cancer: brain tumour (50%), haematological (27.42%), other types of cancer (22.58%)	Brief psychosocial intervention for quality of life (problem-solving, emotional management, etc.) + Usual care	Individual (face-to-face)	5 sessions (1 per week) 30–90 min/session + 5 follow-up calls (1 per week)	Social Worker	Usual care (includes advice and financial support)	5 weeks	1 month	Quality of life (CQOLC)
Sahler et al. (2002), USA and Israel	Hospital	Mothers of children with cancer *N* = 92 (GI: 50; GC: 42) Females: 100% Mean age (years) ± SD: 35.35 ± 6.64 Age range: N.R.	*N* = 92 (GI: 50; GC: 42) Females: N.R. Mean age (years) ± SD: 8.38 ± 5.57 Type of cancer: leukaemia (47.8%), lymphoma (12%), brain tumour (15.2%), other types of cancer (25%)	Problem-solving training program + Usual care	Individual (face-to-face)	8 sessions (1 per week) 60 min/session	Psychologist	Usual care (includes an initial psychosocial assessment by a mental health professional, with referral to psychiatric/psychological, social work or child development support intervention as needed)	8 weeks	3 months	Coping (SPSI-C) Negative mood (POMS)
Sahler et al. (2005), USA and Israel	Hospital	Mothers of children with cancer *N* = 430 (GI: 217; GC: 212) Females: 100% Mean age (years) ± SD: 35.5 ± N.R. Age range: N.R.	*N* = 429 (GI: 217; GC: 212) Females: 49% Mean age (years) ± SD: 7.6 ± N.R. Type of cancer: leukaemia (42.42%), brain tumour (14.92%), lymphoma no Hodgkin (7.93%), Hodgkin’s disease (4.43%), other types of cancer (29.61%)	Problem-solving training program + Usual care	Individual (face-to-face)	8 sessions (1 per week) 60 min/session	N.R.	Usual care (includes an initial comprehensive assessment by a mental health professional within the first few days of diagnosis. Social work, psychological, psychiatric, or other intervention is provided or recommended as needed)	8 weeks	6 months	Coping (SPSI-R) Negative mood (POMS) Depression (BDI) Post-traumatic stress (IES)
Salem et al. (2021), Denmark	Hospital and Home	Parents of children with cancer *N* = 204 (GI: 94; GC: 110) Females: 50% Mean age (years) ± SD: 36.5 ± N.R. years Age range: N.R.	*N* = 204 (GI: 94; GC: 110) Females: 21.6% Mean age (years) ± SD: 4 ± N.R. years Age range: 0–6 Type of cancer: leukaemia or lymphoma (50%), brain tumour (15%), other types of cancer (35%).	FAMily-Oriented Support (FAMOS) intervention (normalising thoughts related to cancer, goal setting and problem-solving and family communication and understanding)	Individual (face-to-face)	5 sessions for parents (N.R. frequency) 60–90 min/session	Psychologists with experience in cognitive-behavioural therapy	Usual psychosocial care (outpatient medical follow-up and management of late effects)	24 weeks	6 months	Post-traumatic stress (HTQ-17) Anxiety (SCL-90-R) Depression (SCL-90-R)
Shakiba et al. (2020), Iran	Hospital	Mothers of children with cancer *N* = 100 (GI: 50; GC: 50) Females: 100% Mean age (years) ± SD: 34.3 ± 6.42 Age range: N.R.	*N* = 100 (GI: 50; GC: 50) Females: 44% Mean age (years) ± SD: 5.4 ± 2.89 Type of cancer: leukaemia (88.5%), other types of cancer (11.5%)	Cognitive-emotional intervention (progressive muscle relaxation, deep breathing, emotional management, cognitive restructuring, mindfulness, etc.)	Group (face-to-face)	5 sessions (2 per week) 100–120 min/session	Psychiatric and oncology nurse supervised by a psychological counsellor	Usual care (routine nursing care provided by the chemotherapy department)	3 weeks	–	Post-traumatic stress (PCL) Resilience (PTGI)
Shekarabi-Ahari et al. (2012), Iran	Hospital	Mothers of children with cancer *N* = 20 (GI:10; GC: 10) Females: 100% Mean age (years) ± SD: 33.35 ± 5.31 Age range: N.R.	*N* = 20 (GI:10; GC: 10) Females: 45% Mean age (years) ± SD: 6.15 ± 4.04 Age range: N.R.	Hope Therapy using Cognitive-Behavioural Therapy techniques (psychoeducation, cognitive distortions, and self-monitoring)	Group (face-to-face)	8 sessions (1 per week) 120 min/session	Therapist (unspecified)	No intervention	8 weeks	–	Depression (BDI) Hope (SHS)
Stehl et al. (2009), USA	Hospital	Parents of children with cancer *N* = 152 (GI: 76; GC: 76) Females: 50% Median age: 39 years	*N* = 152 (GI: 76; GC: 76) Females: 46.1% Median age: 6 years Type of cancer: leukaemia (55.3%), solid tumour (25%), brain tumour (19.7%)	Adapted cognitive-behavioural therapy and family therapy program + Usual care	Individual (face-to-face)	3 sessions (depending on family availability) 45 min/session	Several psychologists and a nurse with specific training in the program	Usual care (routine psychosocial care that included the assignment of a social worker, who provided resources and information about diagnosis and treatment and offered support. Psychologists were available by referral to address behavioural concerns of the child and family)	N.R.	–	Post-traumatic stress (IES) Anxiety (STAI-S)
Streisand (2000), USA	Hospital	Mothers of children with cancer undergoing bone marrow transplantation *N* = 22 (GI: 11; GC: 11) Females: 100% Mean age (years) ± SD: 36.8 ± 6.85 Age range: N.R.	*N* = 22 (GI: 11; GC: 11) Females: 22.7% Mean age (years) ± SD: 8.7 ± 9.7 Type of cancer: N.R.	Intervention based on the stress inoculation model (education, relaxation, communication)	Individual (face-to-face)	1 session 90 min	Psychologist	Usual care (unspecified)	N.A.	–	Stress (DSI)
Tsitsi et al. (2017, 2020), Cyprus and Greece	Hospital and Home	Parents of children with cancer *N* = 54 (GI: 29; GC: 25) Females: N.R. Mean age (years) ± SD: 40.58 ± 6.3 Age range: N.R.	*N* = 54 (GI: 29; GC: 25) Females: N.R. Mean age (years) ± SD: 9.16 ± 4.88 Type of cancer: leukaemia (50%), lymphoma (26.4%), sarcoma (9.4%), brain tumour (3.8%), nephroblastoma (1.9%), ectodermal tumour (1.9%), other types of cancer (5.7%)	Progressive Muscle Relaxation and Guided Imagery Training + Usual care	Individual (face-to-face)	3 sessions (1 per week) 25 min/session + daily practice for 3 weeks	Psychologist + nurse	Usual care (standard psychological support provided by the doctor, nurses and psychologist of the oncology unit)	3 weeks	–	Anxiety (HAM-A) Negative mood (POMS) Coping (WofCC)

Studies with psychological interventions in both children and their parents											
Study (year) country	Setting	Participants		Intervention				Control	Duration	Follow up	Outcomes (scale)
		Characteristics of parents	Characteristics of children eligible for PPC	Content	Type (format)	Number of sessions (frequency) and duration (min)	Provider				
Cho et al. (2023), Akard et al. (2021a), Akard et al. (2021b), USA	Home (Hospice)	Both parents N = 150 (IG: 75; CG: 75) Females: 93% Age mean (years) ± SD: N.R. Age range: N.R.	Children with advanced cancer N = 150 (GI: 37; GC: 61) Females: 57.6% Mean age (years) ± SD: 10 (N.R.) Age range: 7–17 years: Type of cancer: N.R.	Legacy-making through and storytelling	Individual (online-Website)	N.R. Number of sessions (2 weeks) N.R. min/session	Self-administered (online) Storytelling development: Nurses trained in PPC	Usual care (unspecified)	4 weeks	2 months	Parents: Coping (RSQ) Children: Coping (RSQ) Quality of life (PedsQL)
Ebrahimi (2019), Iran	Hospital	Mothers N = 32 (GI: 16; GC: 16) Females: 100% Mean age (years) ± SD: 37.56 ± 6.19 Age range: N.R.	Children with cancer N = 32 (GI: 16; GC: 16) Females: N.R. Mean age (years) ± SD: 9.19 ± 1.15 Age range: N.R. Type of cancer: N.R.	Filial therapy (parent–child play therapy)	Group (face-to-face)	10 sessions (1 per week) 120 min/session	Therapist (unspecified)	No intervention	10 weeks	–	Mothers: Depression (DASS-21) Anxiety (DASS-21) Stress (DASS-21) Children: Depression (CDI)

CG, Control group; IG, Intervention group; N.A., Not applicable; N.R., Not reported; BAI, Beck Anxiety Inventory; BDI, Beck’s Depression Inventory; BFI, Brief Fatigue Inventory; BHS, Beck Hopelessness Scale; CD-RISC, Conner-Davidson Resilience Scale; CDI, Children’s Depression Inventory; CHIP, Coping Health Inventory for Parents; CMCC, Care of My Child with Cancer Scale; COPE, Coping Orientation to Problems Experienced inventory; CQOLC, Caregiver Quality of Life Index-Cancer; DASS-21, Depression Anxiety Stress Scales; DSI, Daily Stress Inventory; DT-P, Distress Thermometer for Parents; GAD-7, The General Anxiety Disorder 7-item Scale; GHQ, Goldberg General Health Questionnaire; HAM-A, Hamilton Anxiety Questionnaire; HTQ-17, 17-item Harvard trauma questionnaire; IES, Impact of Event Scale; KPD, Kessler Psychological Distress scale; MHI, Mental Health Inventory; MLQ, Meaning in Life Questionnaire; OK-Q, Op Koers Questionnaire; PBS, Parent Behavior Scale; PHQ-9, Patient Health Questionnaire-9; PCL, Posttraumatic Stress Disorder Checklist; PECI, Parent Experience of Childhood Illness scale; PedsQL, Paediatric Quality of Life Inventory; POMS, Profile of Mood States; PROMIS, Patient-reported Outcomes Measurement Information System; PSQI, Pittsburgh Sleep Quality Inventory; PSS, Perceived Stress Scale; PTGI, Posttraumatic Growth Inventory; PWBS, Ryff’s Psychological Well-being Scale; RSQ, Response to Stress Questionnaire; SCL-90-R, Symptom Checklist-90-Revised; SCS, Stress Coping Scale; SCQ, Styles of Coping Questionnaire; SF-6D, Short Form of the 6-Dimension Health Survey; SF-12, The Short Form 12-item Health Survey; SHS, Snyder Hope Scale; SPSI-C, Social Problem-Solving Inventory-Cancer; SPSI-R, Social Problem-Solving Inventory-Revised; SRDS, Self-Rating Depression Scale; SSER-Q, Situation-Specific Emotional Reaction Questionnaire; STAI-S, State–Trait Anxiety Inventory; SWLS, Satisfaction with Life Scale; WofCC, Ways of Coping Checklist; WQ, Walsh questionnaire.

The psychological interventions evaluated were heterogeneous, often implemented as multicomponent interventions, with cognitive-behavioural therapy being the most common, alongside techniques such as progressive muscle relaxation, deep breathing, guided imagery, mindfulness, acceptance and commitment therapy, dignity therapy, play therapy, and filial therapy. Interventions were delivered individually or in groups, in-person or virtually, and could be part of multicomponent psychosocial care.

### 3.4 Effects of psychological interventions for parents of children susceptible to PPC

None of the included studies reported adverse effects associated with the psychological interventions. For each outcome measure at the post-intervention time point, a subgroup analysis based on the evaluated population (both parents or mothers only) is provided in the Supplementary material.

#### 3.4.1 PPC-specific oncology settings

##### 3.4.1.1 Coping and health-related quality of life

The only study explicitly conducted within a PPC setting, reported across multiple publications (Cho et al., 2023; Akard et al., 2021a,b), evaluated the effects of a single-session legacy-making intervention among children with advanced cancer and their parents. The study assessed coping outcomes in both children and parents, finding no statistically significant differences between the intervention and control groups 2 months after the intervention for either children (Akard et al., 2021a) or parents (Cho et al., 2023). In addition, children’s health-related quality of life was evaluated, with no significant differences observed between groups 2 months after the intervention (Akard et al., 2021b).

#### 3.4.2 Broader paediatric oncology settings

##### 3.4.2.1 Anxiety

Eighteen studies assessed anxiety symptoms in parents of children with cancer after intervention (Bahrami et al., 2020; Ebrahimi et al., 2019; Joosten et al., 2024; Kazak et al., 2005; Manne et al., 2016; Marsland et al., 2013, 2020; Ozturk and Katikol, 2024; Pouraboli et al., 2019; Stehl et al., 2009; Tsitsi et al., 2017; Cernvall et al., 2015; Delavari et al., 2014; Gholamian et al., 2021; Hoekstra-Weebers et al., 1998; Jay and Elliott, 1990; Phiri et al., 2023; Salem et al., 2021) and a MA could be conducted with 17 of them for post-intervention evaluation (Bahrami et al., 2020; Ebrahimi et al., 2019; Joosten et al., 2024; Kazak et al., 2005; Manne et al., 2016; Marsland et al., 2013, 2020; Ozturk and Katikol, 2024; Pouraboli et al., 2019; Stehl et al., 2009; Tsitsi et al., 2017; Cernvall et al., 2015; Delavari et al., 2014; Gholamian et al., 2021; Phiri et al., 2023; Salem et al., 2021; Hoekstra-Weebers et al., 1998) (*N* = 1,289) and at 3, 6, and 12-month follow-up assessments.

The studies included in the MA encompassed a wide range of psychological interventions, reflecting substantial heterogeneity in their theoretical foundations and formats. Overall, the interventions were: emotional regulation training based on Gross’s psychological model (Bahrami et al., 2020), online guided self-help program based on cognitive-behavioural therapy (Cernvall et al., 2015), logotherapy (Delavari et al., 2014), filial therapy (Ebrahimi et al., 2019), peer self-help group program (Gholamian et al., 2021), an online active coping skills program based on cognitive-behavioural and acceptance and commitment techniques (Joosten et al., 2024), an adapted cognitive-behavioural therapy and family therapy program (Kazak et al., 2005; Stehl et al., 2009; Salem et al., 2021), brief cognitive-behavioural therapy (Manne et al., 2016), a multimodal intervention for stress management based on cognitive-behavioural techniques (Marsland et al., 2013, 2020), an online relaxation program based on cognitive-behavioural techniques and mindfulness (Ozturk and Katikol, 2024), psychoeducational intervention (Phiri et al., 2023), a guided intervention focused on cognitive-behavioural techniques and psychoeducation (Hoekstra-Weebers et al., 1998), progressive muscle relaxation training (Pouraboli et al., 2019) and progressive muscle relaxation and guided imagery training (Tsitsi et al., 2017). The duration of the interventions ranged from 3 to 12 sessions.

**Figure 2:**
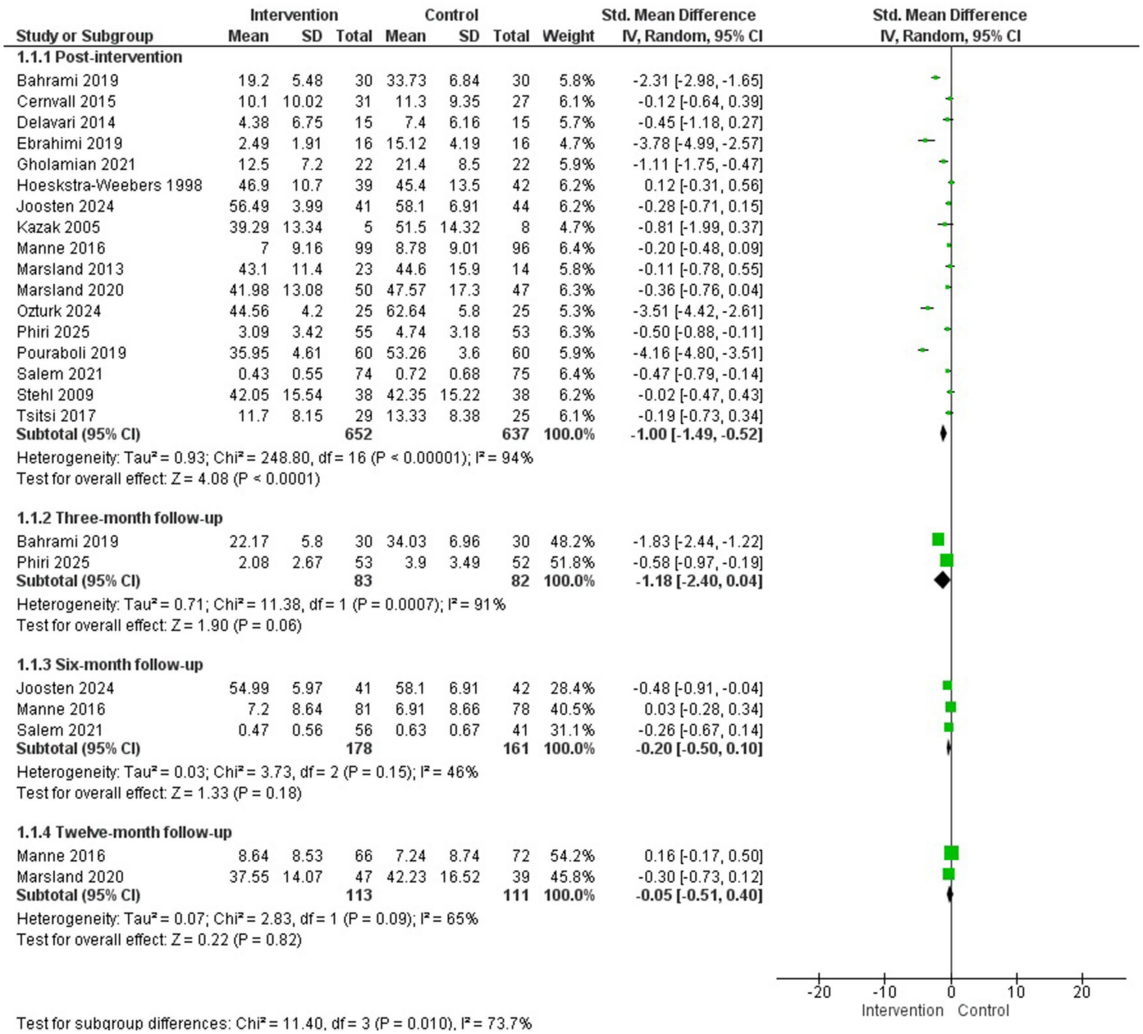
Anxiety.

The results of the MA showed a statistically significant difference in favour of the intervention group compared to the control group in the reduction of anxiety symptoms after the intervention (*g* = −1.00; 95% CI: −1.49, −0.52; I^2^ = 94%; *p* < 0.00001) but not at 3, 6, or 12-month follow-up assessments (Figure 2). Regarding publication bias, the Egger’s test was not significant (see Supplementary Figure S2). A subgroup analysis by population (studies including only mothers versus studies including both parents) found no statistically significant difference between subgroups post-intervention (see Supplementary Figure S3).

Additionally, of the studies that could not be included in the MA for this outcome measure, Jay and Elliott (1990) observed no statistically significant differences between groups after a stress inoculation session to improve anxiety symptoms in parents of children with cancer during invasive medical procedures (bone marrow biopsy or lumbar puncture). Meanwhile, Cernvall et al. (2017), a follow-up to Cernvall et al. (2015), found statistically significant differences in favour of the intervention group 1 year after the intervention (*p* < 0.001).

##### 3.4.2.2 Depression

Thirteen studies evaluated symptoms of depression in parents of children with cancer (Sahler et al., 2005; Shekarabi-Ahari et al., 2012; Marsland et al., 2013; Delavari et al., 2014; Cernvall et al., 2015; Manne et al., 2016; Cernvall et al., 2017; Ebrahimi et al., 2019; Marsland et al., 2020; Luo et al., 2021a; Salem et al., 2021; Park et al., 2023; Phiri et al., 2023; Joosten et al., 2024) and a MA could be conducted with 10 of them for post-intervention evaluation (Marsland et al., 2013; Delavari et al., 2014; Cernvall et al., 2015; Manne et al., 2016; Ebrahimi et al., 2019; Marsland et al., 2020; Luo et al., 2021a; Salem et al., 2021; Phiri et al., 2023; Joosten et al., 2024) (*N* = 900) and 3, 6, and 12-month follow-up assessments.

The psychological interventions included in the MA were highly heterogeneous and encompassed a wide range of approaches, including: an online guided self-help program based on cognitive-behavioural therapy (Cernvall et al., 2015), logotherapy (Delavari et al., 2014), filial therapy (Ebrahimi et al., 2019), an online active coping skills program based on cognitive-behavioural and acceptance and commitment techniques (Joosten et al., 2024), an online resilience training program (Luo et al., 2021a), brief cognitive-behavioural therapy (Manne et al., 2016), an adapted cognitive-behavioural therapy and family therapy program (Salem et al., 2021), psychoeducational intervention (Phiri et al., 2023), and a multimodal stress management intervention based on cognitive-behavioural techniques (Marsland et al., 2013, 2020). The duration of the interventions ranged from 3 to 12 sessions.

**Figure 3:**
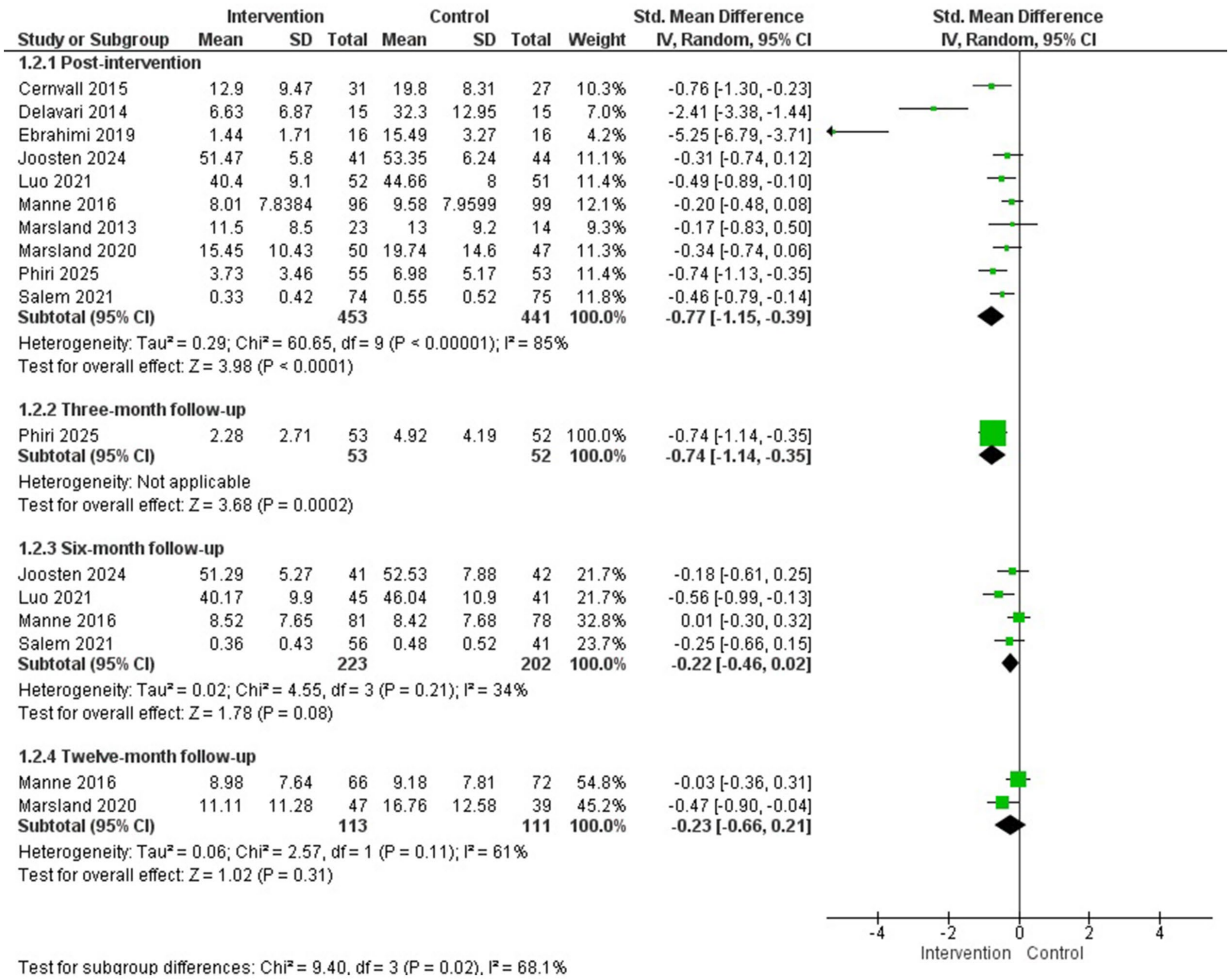
Depression.

The results of the MA showed a statistically significant difference in favour of the intervention group compared to the control group in the reduction of depression symptoms after the intervention (*g* = −0.77; 95% CI: −1.15, −0.39; I^2^ = 85%; *p* < 0.0001) and at 3 month follow-up (1 study) (*g* = −0.74; 95% CI: −1.14, −0.35; *p* = 0.0002), but not at 6- or 12-month follow-up (Figure 3). Regarding publication bias, the Egger’s test was not significant (see Supplementary Figure S4). A subgroup analysis by population (studies including only mothers versus studies including both parents) found no statistically significant difference between subgroups (see Supplementary Figure S5).

Additionally, among the four studies that could not be included in the MA due to insufficient data, three reported statistically significant improvements in favour of the intervention group (*p* < 0.001). These effects were observed following a problem-solving training program (8 sessions) (Sahler et al., 2005) and Hope Therapy based on cognitive-behavioural techniques (8 sessions) (Shekarabi-Ahari et al., 2012), as well as at 6-month (Sahler et al., 2005) and 12-month follow-up (Cernvall et al., 2017). In contrast, the study by Park et al. (2023) found no statistically significant differences between groups in depressive symptoms following an online family resilience program (4 sessions).

Regarding child outcomes, Ebrahimi et al. (2019), reported a significant reduction in depressive symptoms favoring the filial therapy group (10 sessions) compared with the control group at post-intervention (*p* < 0.001).

##### 3.4.2.3 Health-related quality of life

An MA was conducted including four of the five studies that evaluated health-related quality of life of parents of children with cancer after the intervention and 1, 6, and 12-month follow-up (Safarabadi-Farahani et al., 2016; Marsland et al., 2020; Luo et al., 2021a; Jin et al., 2023) (*N* = 302).

The psychological interventions evaluated were acceptance and commitment therapy (Jin et al., 2023), a multimodal stress management intervention based on cognitive-behavioural techniques (Marsland et al., 2020), an online resilience training program (Luo et al., 2021a), and a brief psychosocial intervention based on problem-solving, emotional management, etc. (Safarabadi-Farahani et al., 2016). The duration of the interventions ranged from 4 to 12 sessions.

**Figure 4:**
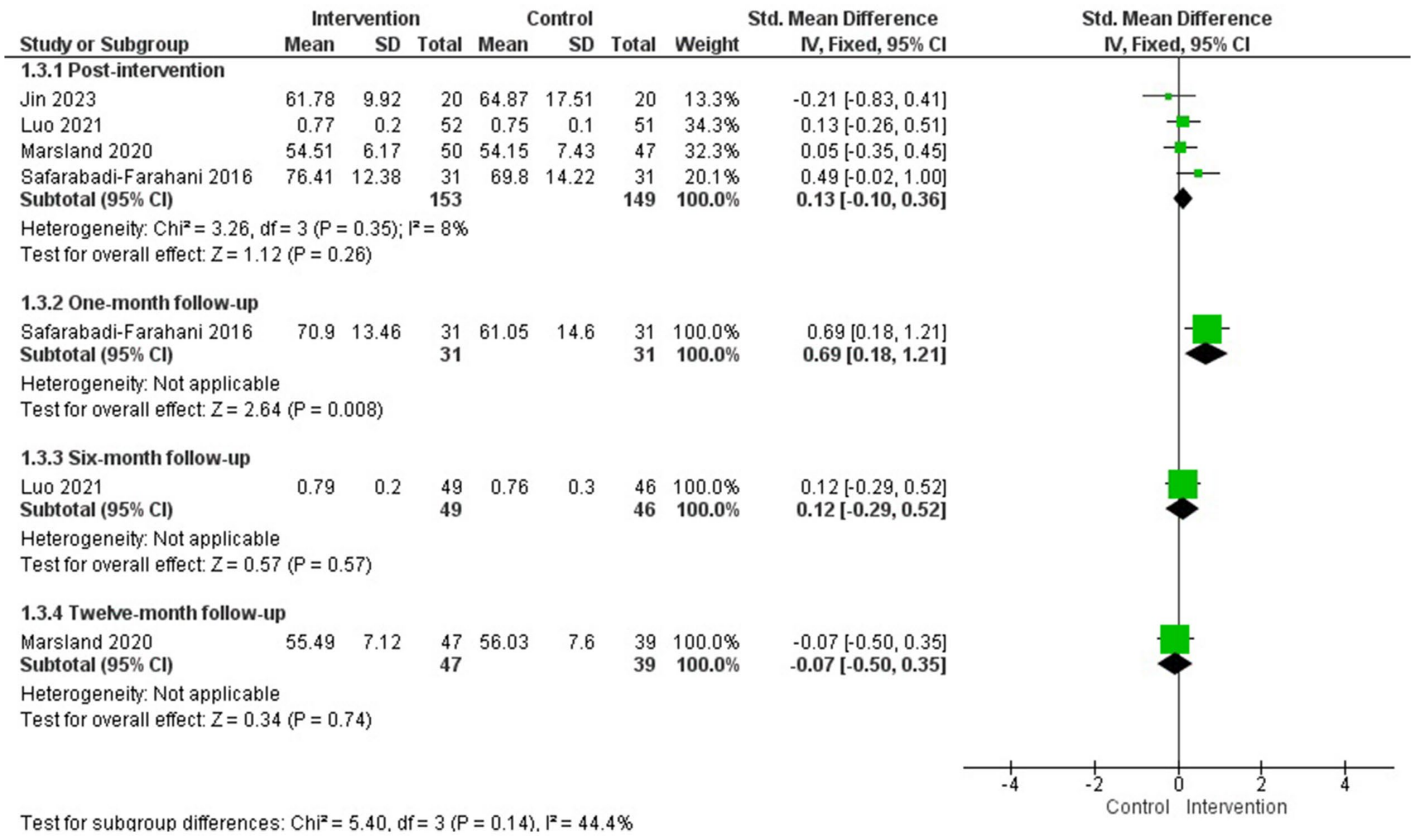
Health-related quality of life.

The MA showed no statistically significant differences between groups in health-related quality of life improvement at post-intervention or at 6- or 12-month follow-up (one study at each follow-up time point). However, a statistically significant difference in favour of the intervention group was observed at 1-month follow-up (one study) (*g* = 0.69; 95% CI: 0.18, 1.21; *p* = 0.008) (Figure 4). Marsland et al. (2020) was the only one involving only mothers, and when it was removed from the MA the results remained non-significant (see Supplementary Figure S6). Phiri et al. (2025), which could not be included in the MA, also found no statistically significant differences between groups post-intervention and at 3-month follow-up.

##### 3.4.2.4 Posttraumatic stress

Eight studies assessed posttraumatic stress symptoms in parents of children with cancer (Sahler et al., 2005; Stehl et al., 2009; Mullins et al., 2012; Marsland et al., 2013, 2020; Cernvall et al., 2015; Manne et al., 2016; Shakiba et al., 2020), and a MA was available for seven of them for post-intervention evaluation (Stehl et al., 2009; Marsland et al., 2013, 2020; Cernvall et al., 2015; Manne et al., 2016; Shakiba et al., 2020; Salem et al., 2021) (*N* = 724) and 3, 6, and 12-month follow-up.

The psychological interventions evaluated were: an online guided self-help program based on cognitive-behavioural therapy (Cernvall et al., 2015), an adapted cognitive-behavioural therapy and family therapy program (Stehl et al., 2009; Salem et al., 2021), brief cognitive-behavioural therapy (Manne et al., 2016), a multimodal stress management intervention based on cognitive-behavioural techniques (Marsland et al., 2013, 2020), a problem-solving training program (Sahler et al., 2005), and cognitive-emotional training (Shakiba et al., 2020). The duration of the interventions ranged from 3 to 12 sessions.

**Figure 5:**
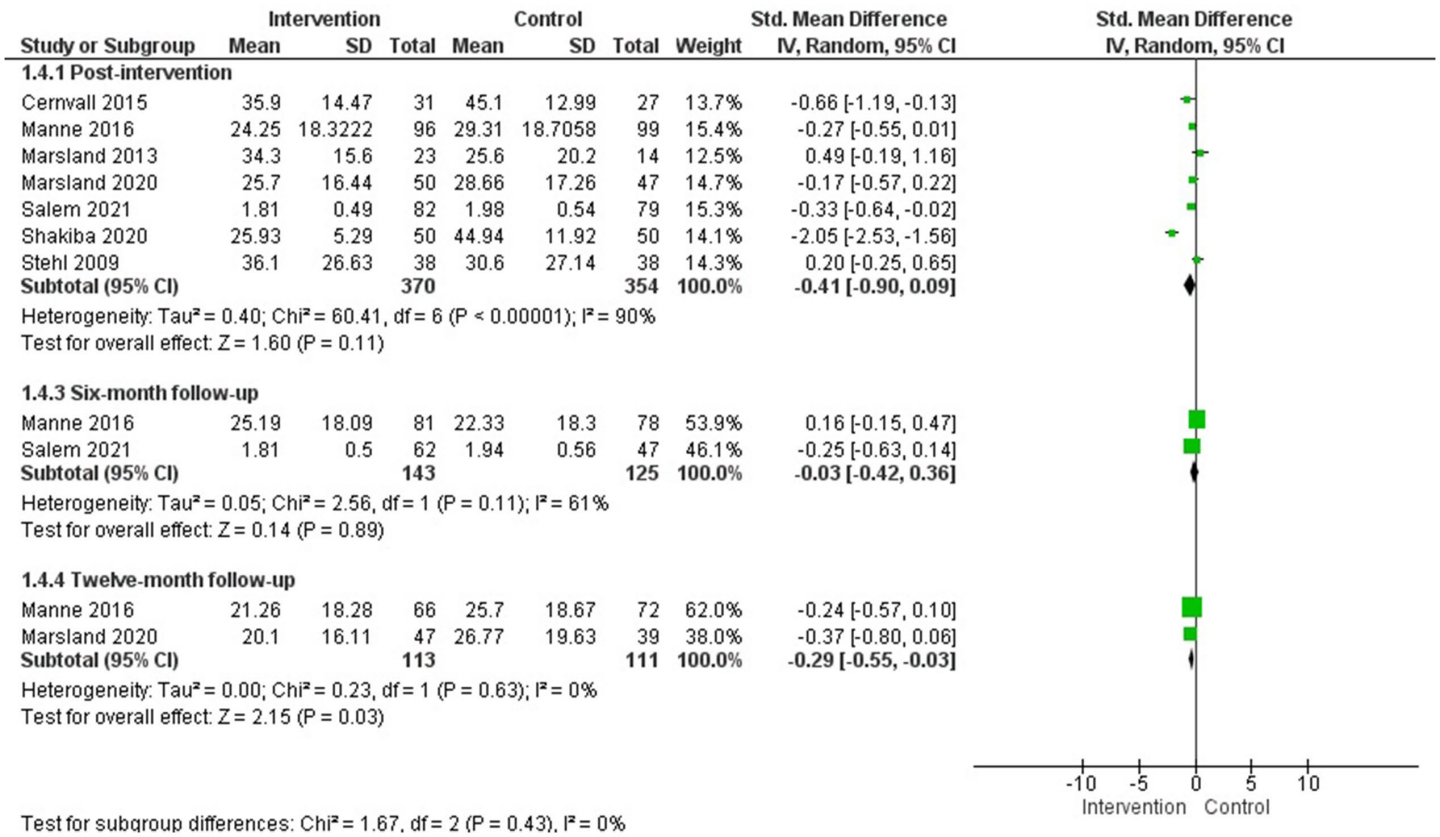
Posttraumatic stress symptoms.

The MA showed a statistically significant difference in favour of the intervention group compared with the control group in the reduction of posttraumatic stress symptoms at 12-month follow-up (*g* = −0.29; 95% CI: −0.55, −0.03; I^2^ = 0%; *p* = 0.03), but not at post-intervention or at 6-month follow-up (Figure 5). A subgroup analysis by population (studies including only mothers versus studies including both parents) found no statistically significant differences between groups (see Supplementary Figure S7).

Regarding the studies that could not be included in the MA due to insufficient data, two reported statistically significant improvements in favour of the intervention group compared to the control group both after the intervention (Sahler et al., 2005; Mullins et al., 2012) and at 6-month follow-up (*p* < 0.001) (Sahler et al., 2005).

##### 3.4.2.5 Psychological distress

Four studies assessed psychological distress symptoms in parents of children with cancer (Jay and Elliott, 1990; Latifi et al., 2021; Jin et al., 2023; Joosten et al., 2024) and a MA could be conducted with two of them for post-intervention assessment (*N* = 125) (Jin et al., 2023; Joosten et al., 2024) as well as at 6-month follow-up.

The psychological interventions included in the MA were acceptance and commitment therapy (Jin et al., 2023) and an online active coping skills program based on cognitive-behavioural and acceptance and commitment techniques (Joosten et al., 2024). The duration of the interventions were 4 and 6 sessions, respectively.

**Figure 6:**
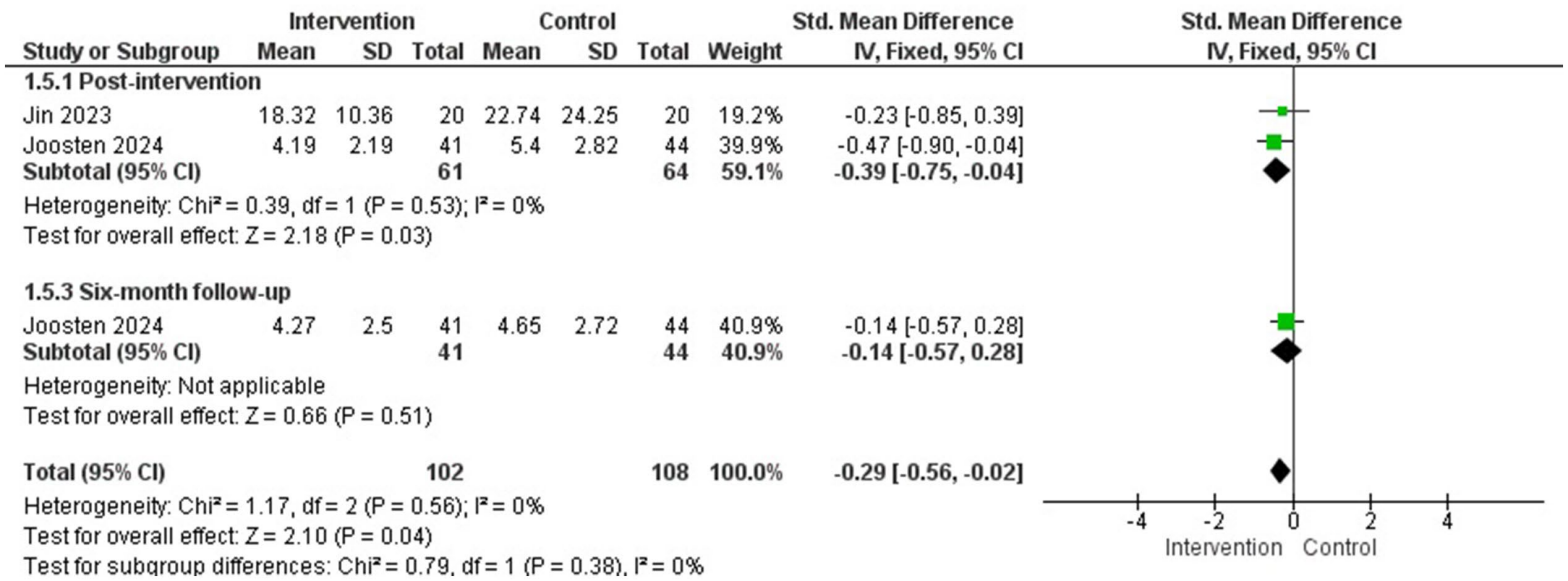
Psychological distress symptoms.

The results of the MA showed a statistically significant difference in favour of the intervention group versus the control group in the reduction of psychological distress symptoms after the intervention (*g* = −0.39; 95% CI: −0.75, −0.04; I^2^ = 0%; *p* = 0.03) but not at 6-month follow-up (1 study) (Figure 6). No subgroup analyses were performed.

Regarding the studies that did not provide the necessary data to be included in the MA for this outcome measure, the results are heterogeneous. On the one hand, the study by Jay and Elliott (1990) (*N* = 72), which evaluated a stress inoculation session, did not observe statistically significant differences between groups. On the other hand, the study by Latifi et al. (2021) that evaluated a 5-session cognitive-emotional intervention found a statistically significant reduction in favour of the intervention group compared to the control group at the 4-month follow-up (*p* < 0.001).

##### 3.4.2.6 Stress

An MA was conducted with five studies assessing stress symptoms in parents of children with cancer at post-intervention and 12-month follow-up (Streisand, 2000; Marsland et al., 2013, 2020; Kaboudi et al., 2018; Ebrahimi et al., 2019) (*N* = 246).

The psychological interventions evaluated were child-centred therapy delivered to parents (Ebrahimi et al., 2019), a resilience training program (Kaboudi et al., 2018), an intervention based on the stress inoculation model (Streisand, 2000), and a multimodal stress management intervention based on cognitive-behavioural techniques (Marsland et al., 2013, 2020). The duration of the interventions ranged from 1 to 12 sessions.

**Figure 7:**
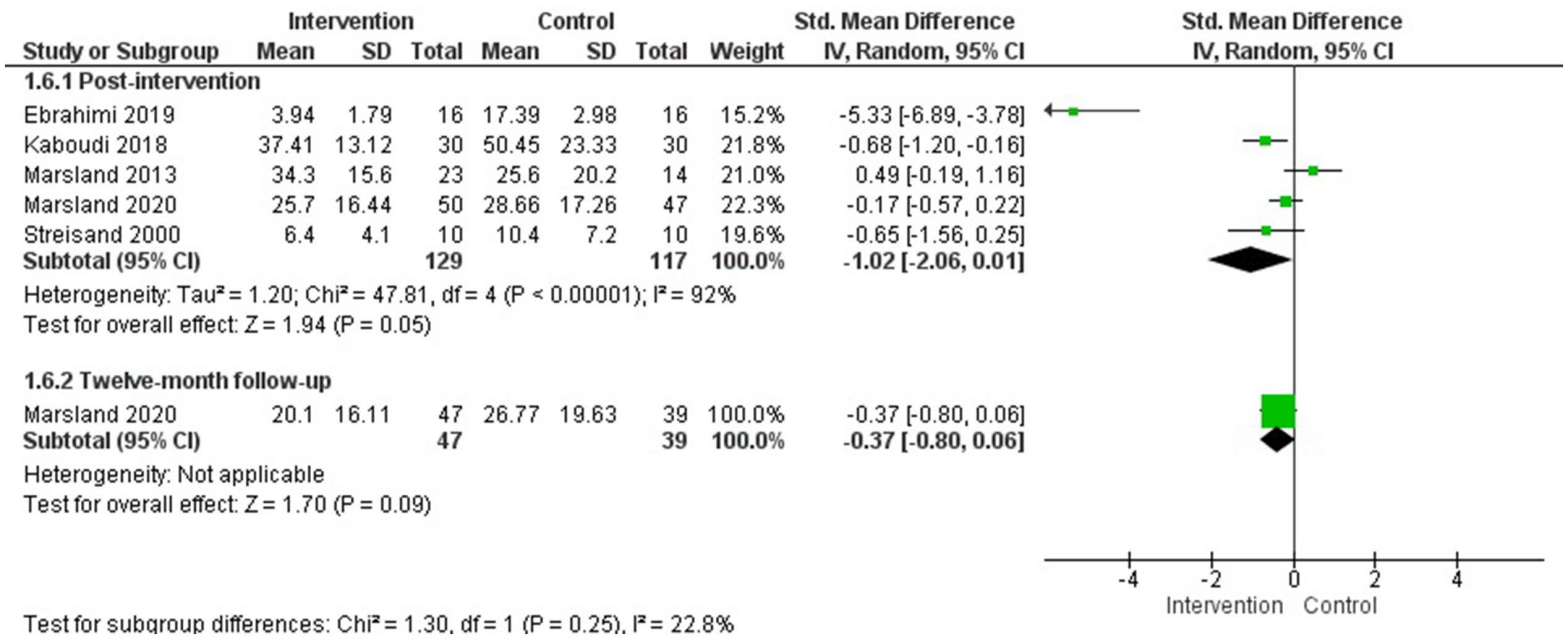
Stress symptoms.

The MA results showed no statistically significant differences between groups in stress reduction after the intervention or at 12-month follow-up (1 study) (Figure 7). Marsland et al. (2013) was the only study including both parents, and when it was excluded from the MA the results change to a statistically significant difference in favour of the intervention group compared with the control group at post-intervention (*g* = −1.45; 95% CI: −2.70, −0.21; I^2^ = 92%; *p* = 0.02) (see Supplementary Figure S8).

##### 3.4.2.7 Coping

Seven studies evaluated coping in parents of children with cancer (Sahler et al., 2002, 2005; Kaboudi et al., 2018; Hoseinzadeh et al., 2019; Tsitsi et al., 2020; Joosten et al., 2024; Ozturk and Katikol, 2024; Phiri et al., 2025) and an MA was conducted with two of them for post-intervention evaluation (Kaboudi et al., 2018; Ozturk and Katikol, 2024) (*N* = 210).

The psychological interventions included in the MA were an online relaxation program based on cognitive-behavioural techniques and mindfulness (Ozturk and Katikol, 2024) and a resilience training program (Kaboudi et al., 2018). The duration of the interventions were 7 and 9 sessions, respectively.

**Figure 8:**
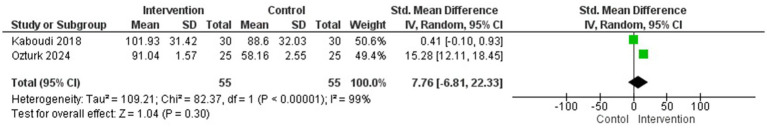
Coping.

The results of the MA showed no significant difference between groups after the intervention in coping improvement (Figure 8). No subgroup analyses were performed.

Additionally, of the studies that could not be included in the MA for this outcome measure, the results are heterogeneous. In two of the included studies, no statistically significant differences were observed between groups after administering an online active coping skills program based on cognitive-behavioural and acceptance and commitment techniques (Joosten et al., 2024), and training in progressive muscle relaxation and guided imagery (Tsitsi et al., 2020). In contrast, Hoseinzadeh et al. (2019) observed statistically favourable results for a resilience-based intervention both after the intervention and at the 3-month follow-up (*p* < 0.001). Phiri et al. (2025) found the same results after a multicomponent group psychoeducational intervention (health education, coping skills training, stress management techniques training and psychological support) (*p* < 0.001). Two studies evaluated problem solving in parents of children with cancer as a coping measure, although they did not provide the data necessary to be combined in an MA. The results showed a statistically significant increase in favour of the intervention group compared to the control group after the intervention (*p* = 0.05) (Sahler et al., 2002, 2005) and at the 3-month follow-up (*p* = 0.008) (Sahler et al., 2002), but not at the 6-month follow-up.

##### 3.4.2.8 Resilience

Six studies evaluated resilience in parents of children with cancer (Jamali et al., 2019; Porter et al., 2019; Shakiba et al., 2020; Luo et al., 2021a; Khosrobeigi et al., 2022; Park et al., 2023), and a MA was conducted with five of them for post-intervention evaluation (Jamali et al., 2019; Porter et al., 2019; Shakiba et al., 2020; Luo et al., 2021a; Khosrobeigi et al., 2022) (*N* = 341) and 2 and 6-month follow-up.

The psychological interventions evaluated in the MA were a peer psychoeducation program focused on resilience (Jamali et al., 2019), compassion therapy (Khosrobeigi et al., 2022), an online resilience training program (Luo et al., 2021a), couples therapy (Porter et al., 2019), and cognitive-emotional training (Shakiba et al., 2020). The duration of the interventions ranged from 5 to 8 sessions.

**Figure 9:**
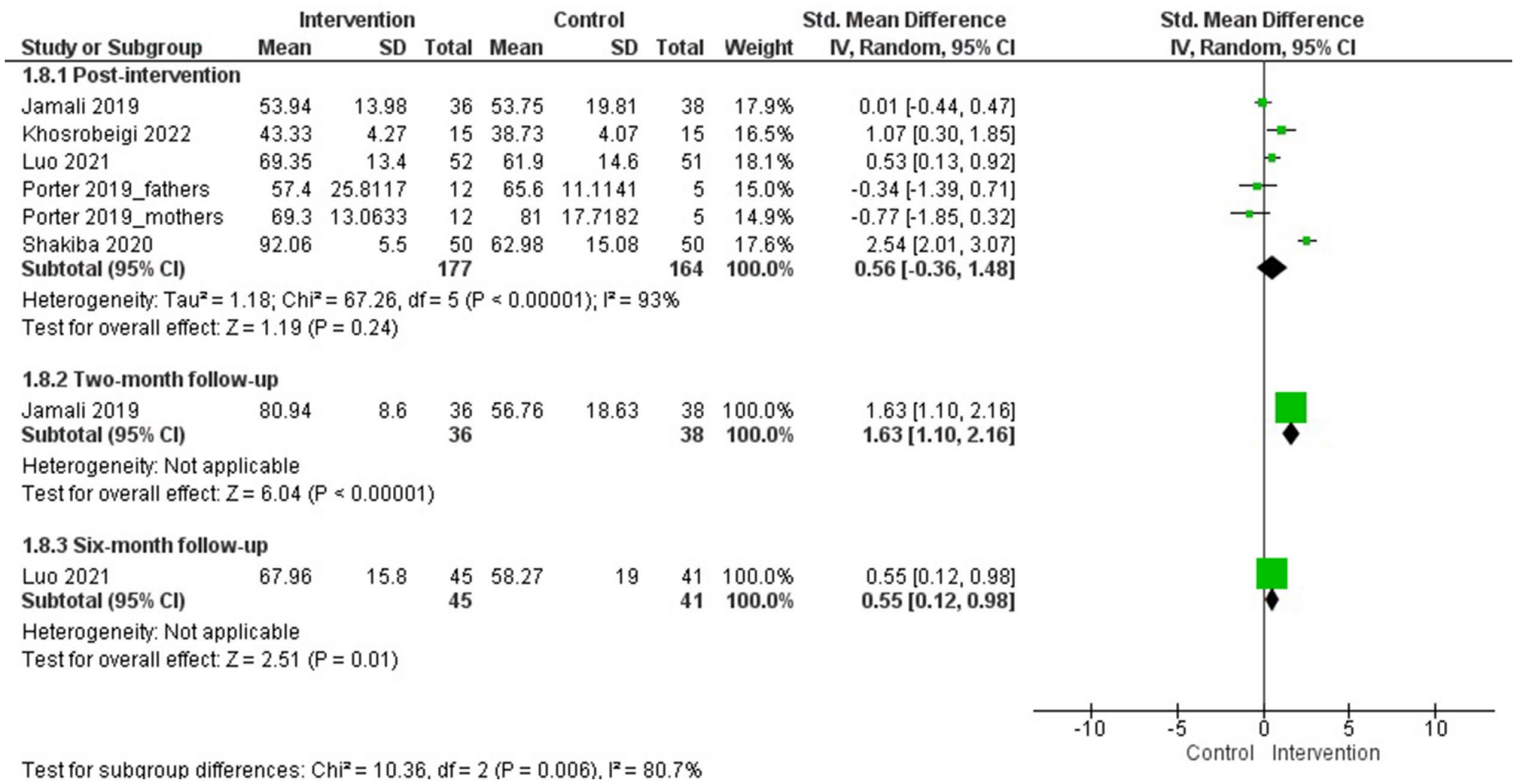
Resilience.

The MA results showed no significant differences between groups post-intervention in improvement resilience, but significant differences were observed at 2-month follow-up (1 study) (*g* = 1.63; IC95%: 1.10, 2.16; *p* < 0.00001) and 6-month follow-up (1 study) (*g* = 0.55; IC95%: 0.12, 0.98; *p* = 0.01) (Figure 9). A subgroup analysis by population (studies including only mothers versus studies including both parents) found no statistically significant difference between subgroups (see Supplementary Figure S9).

Additionally, the study by Park et al. (2023), which did not provide data for inclusion in the MA for this outcome measure, also found statistically significant results in favour of the intervention group after the intervention (*p* = 0.003) and at the 1-month follow-up (*p* < 0.05).

##### 3.4.2.9 Hope

Three studies evaluated hope in parents of children with cancer (Shekarabi-Ahari et al., 2012; Damreihani et al., 2018; Khosrobeigi et al., 2022), and a MA was conducted with two of them (*N* = 60) (Damreihani et al., 2018; Khosrobeigi et al., 2022).

The psychological interventions evaluated in the MA were a task-oriented positive psychology intervention to improve positive abilities and emotions (Damreihani et al., 2018) and compassion therapy (Khosrobeigi et al., 2022). The duration of the interventions were 6 and 8 sessions, respectively.

**Figure 10:**
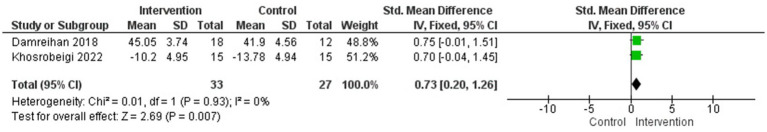
Hope.

The MA results showed a statistically significant difference in favour of the intervention group versus the control group after the intervention in improvement hope (*g* = 0.73; 95% CI: 0.20, 1.26; I^2^ = 0%; *p* = 0.007) (Figure 10). No subgroup analyses were performed.

Additionally, the study by Shekarabi-Ahari et al. (2012), which did not provide the necessary data for inclusion in the MA for this outcome measure, compared cognitive-behavioural-based Hope Therapy (8 sessions) also found statistically significant results in favour of the intervention group versus the control group were observed after the intervention (*p* = 0.001).

##### 3.4.2.10 Psychological well-being

An MA was conducted with two studies evaluating the psychological well-being of parents of children with cancer (*N* = 225) (Manne et al., 2016; Damreihani et al., 2018).

The psychological interventions evaluated were brief cognitive-behavioural therapy (Manne et al., 2016) and a task-oriented positive psychology intervention to improve positive abilities and emotions (Damreihani et al., 2018). The duration of the interventions was 5 and 6 sessions, respectively.

**Figure 11:**
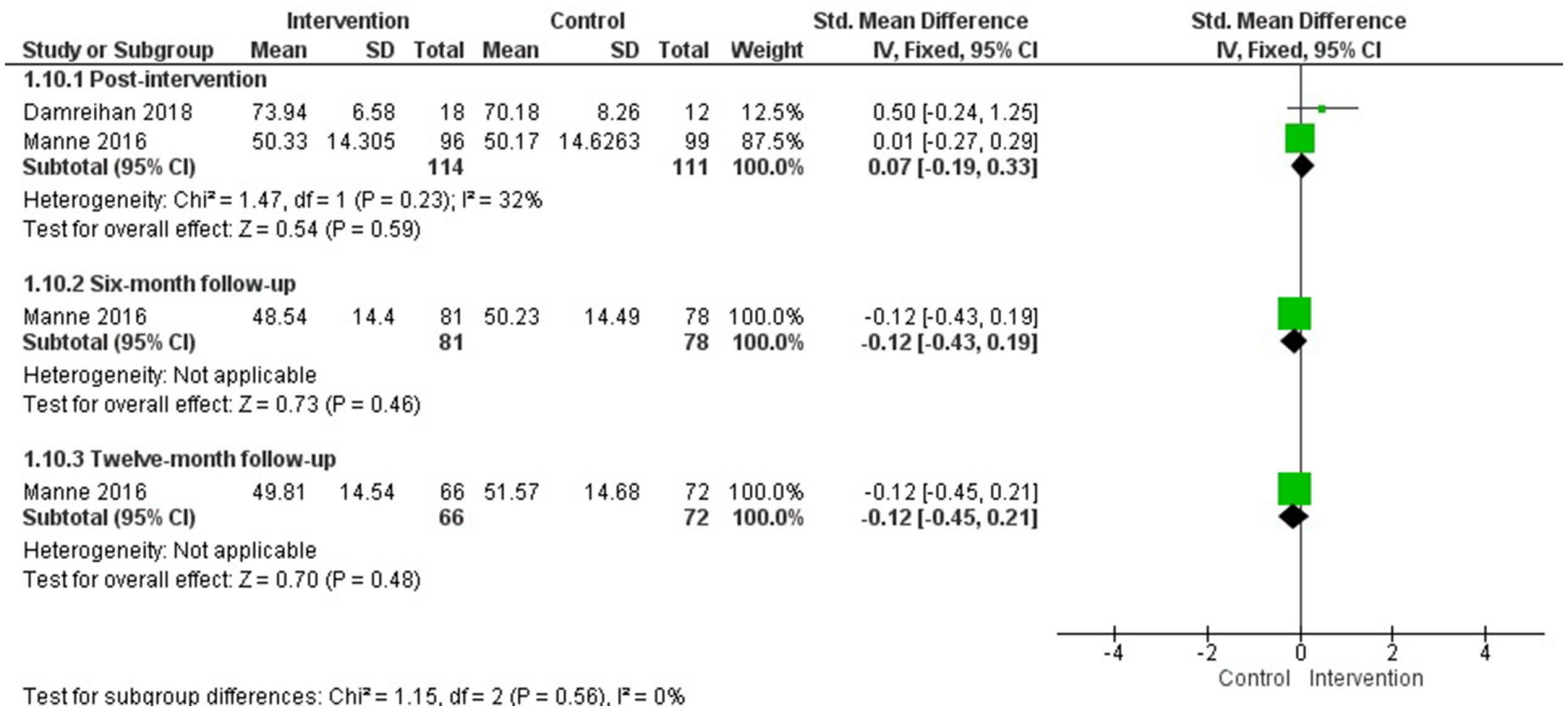
Psychological well-being.

The MA results showed no significant differences between groups in terms of improved psychological well-being post intervention or any follow-up periods (Figure 11). No subgroup analyses were performed.

##### 3.4.2.11 Emotional impact of parent experience of child’s illness

An MA was conducted with two studies evaluating the emotional impact of the experience of a child’s illness on parents of children with cancer (Porter et al., 2019; Jin et al., 2023) (*N* = 82).

The psychological interventions evaluated were acceptance and commitment therapy (Jin et al., 2023) and couples therapy (Porter et al., 2019). The duration of the interventions was 4 and 6 sessions, respectively.

**Figure 12:**
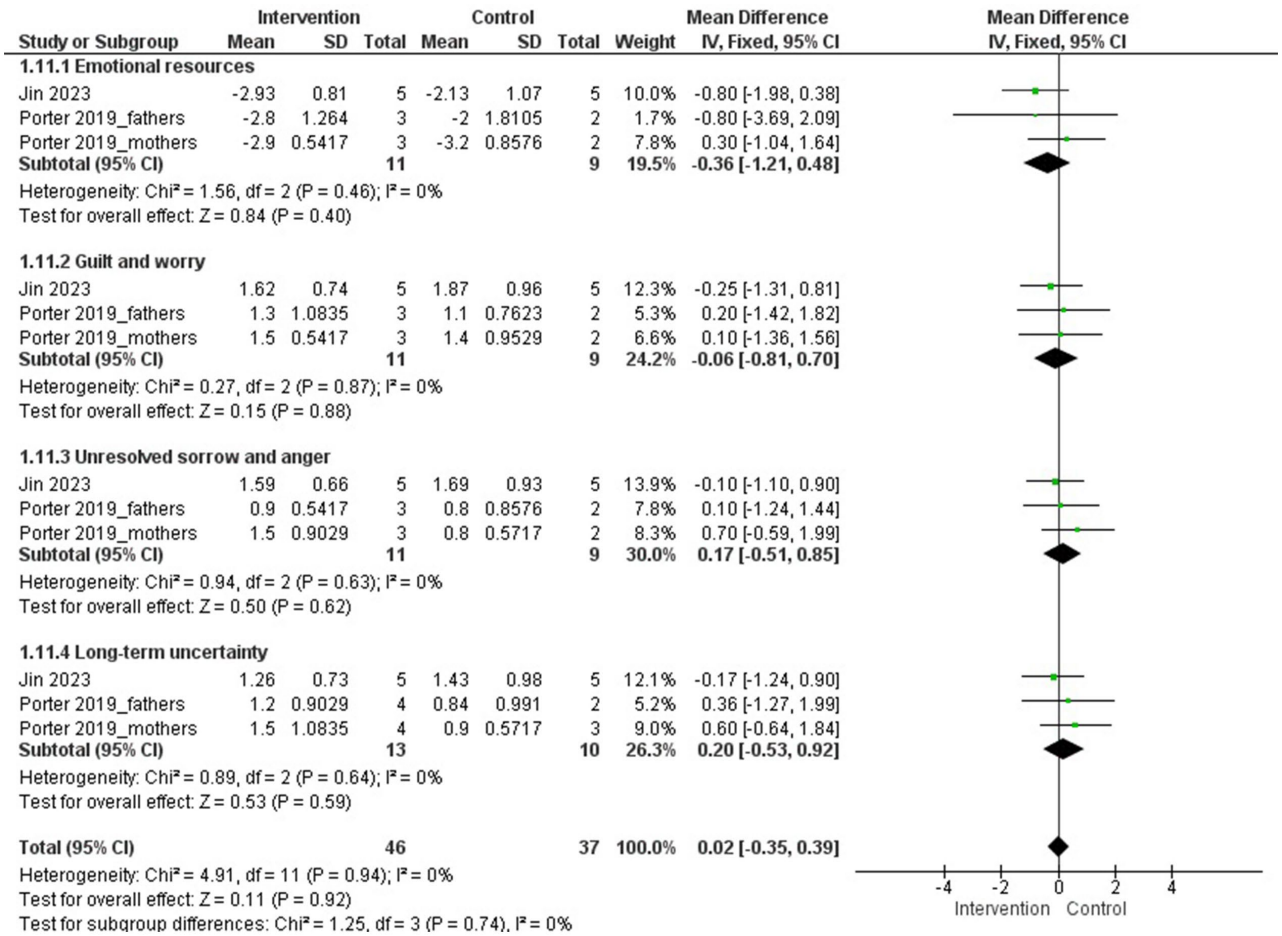
Emotional impact of the experience of a child’s illness.

The MA results showed no statistically significant differences between groups on any of the PECI subscales after the intervention (emotional resources, guilt and worry, unresolved grief and anger, and uncertainty about the long term) (Figure 12). No subgroup analyses were performed.

##### 3.4.2.12 Negative mood

Three studies assessed negative mood in parents of children with cancer using the Profile of Mood States (POMS), although they did not provide combinable data for a MA for this outcome measure. Specifically, the study by Sahler et al. (2002) evaluated an 8-session problem-solving training program in mothers of children with cancer. Results showed a statistically significant improvement in favour of the intervention group compared to the control group post-intervention (*p* = 0.003) and at the 3-month follow-up (*p* = 0.003) in the total POMS score. Sahler et al. (2005) also evaluated a 8-session problem-solving training program versus usual care in mothers of children with cancer, with results showing a statistically significant improvement in favour of the intervention group post-intervention (*p* = 0.001) in the total POMS score. No significant differences were observed at the 6-month follow-up. Finally, the study by Tsitsi et al. (2017) evaluated a 3-session progressive muscle relaxation and guided imagery program with daily home practice progressive muscle relaxation and guided imagery training in parents of children with cancer. Results showed a statistically significant improvement in the intervention group post-intervention in the anxiety/tension subscale (*p* = 0.027), but not in the other subscales (depression, anger/hostility, fatigue, confusion). Between-group comparisons were not reported.

##### 3.4.2.13 Other outcomes

In relation to other outcome measures assessed in the identified studies, at least one study was found that evaluated caregiver burden, meaning and life satisfaction, disease-related emotional reactions, self-perceived general parental mental health, psychiatric symptoms, fatigue, and sleep quality.

The study by Mullins et al. (2012) evaluated a 12-sessions cognitive-behavioural therapy program aimed at improving caregiver burden perception in mothers of children with cancer. Results, measured with the Care of My Child with Cancer Scale (CMCC), showed a statistically significant reduction in favour of the intervention group compared to the control group (*p* = 0.003); however, this effect was not maintained at the 3-month follow-up.

In the study by Damreihani et al. (2018), a 6-sessions task-oriented positive psychology intervention was evaluated to enhance positive capacities and emotions to improve meaning and life satisfaction in mothers of children with cancer. Results, assessed with the Meaning in Life Questionnaire (MLQ) and the Satisfaction with Life Scale (SWLS), showed statistically significant improvement in favour of the intervention group immediately post-intervention and at the 1-month follow-up (*p* < 0.05) for both measures.

The study by Joosten et al. (2024) evaluated a 6-sessions online active coping skills program based on cognitive-behavioural and acceptance and commitment techniques to improve disease-specific emotional reactions (anxiety, sadness or depression, irritability or frustration, shame or discomfort, hope or relief) in parents of children with cancer. Results, measured with the Situation-Specific Emotional Reaction Questionnaire (SSER-Q), showed a statistically significant reduction in the loneliness subscale in favour of the intervention group post-intervention (*p* < 0.05). At the 6-month follow-up, these differences were no longer significant, and no differences between groups were observed in the other subscales (helplessness, positive feelings, and uncertainty) at any time point.

The study by Hoekstra-Weebers et al. (1998) evaluated an 8-sessions guided intervention focused on cognitive-behavioural techniques and psychoeducation to improve self-perceived general mental health and reduce psychiatric symptoms in parents of children with cancer. Results, assessed with the General Health Questionnaire (GHQ) and the Symptom Checklist-90 (SCL-90), showed no statistically significant differences between the intervention and control groups either immediately post-intervention or at the 6-month follow-up for any outcome.

Finally, the study by Pouraboli et al. (2019) evaluated an 8-sessions progressive muscle relaxation training to improve fatigue and sleep quality in parents of children with cancer. Results, measured with the Brief Fatigue Inventory (BFI) and the Pittsburgh Sleep Quality Index (PSQI), showed statistically significant improvements in favour of the intervention group compared to the control group post-intervention (*p* < 0.0001) for both outcomes.

## 4 Discussion

### 4.1 Effectiveness of the interventions

Psychological interventions targeting parents of children with cancer were generally associated with positive effects on psychological outcomes. Our findings align with prior literature demonstrating that psychological interventions focused on parents can meaningfully enhance their mental health and strengthen their resilience (Eccleston et al., 2015; Coughtrey et al., 2018; Law et al., 2019; Luo et al., 2021b; Sánchez-Egea et al., 2019). The evidence base primarily reflects paediatric oncology settings, with only one trial explicitly embedded in PPC. This distinction is important, as findings from oncology-specific populations may not fully generalize to broader PPC contexts.

Results indicated significant short-term improvements in anxiety, depression, psychological distress, and hope (*p* < 0.05) following psychological support interventions. Effects were most consistent for short-term anxiety and depression, whereas evidence for health-related quality of life, coping, and stress outcomes was mixed or largely non-significant. Evidence for sustained effects over longer follow-up periods was limited and inconsistent. Anxiety and depression effects were primarily observed immediately post-intervention, with no consistent maintenance at 6 or 12 months. In contrast, reductions in post-traumatic stress symptoms were reported mainly at longer-term follow-up rather than immediately post-intervention. Narrative findings from a small number of individual studies suggested potential improvements in caregiver burden, life satisfaction, meaning, fatigue, sleep quality, and illness understanding in specific contexts; however, these findings were inconsistent and based on limited evidence. Overall, short-term symptom improvements are more consistent than long-term effects, and sustained effects remain uncertain, potentially indicating the need for booster or stepped-care approaches. Furthermore, there was considerable heterogeneity in intervention types, delivery modes, session duration, and outcome measures across studies. Interventions ranged from single session to multi-session programs, delivered individually, in groups, or online. This substantial heterogeneity likely contributed to variability in observed effects and complicates direct comparison across studies, indicating that pooled effect sizes should be interpreted with caution and underscoring the need for standardized interventions and outcome measures in future research.

### 4.2 Evidence quality and research gaps

Despite these promising results, the quality of the available evidence is limited by substantial methodological heterogeneity, small sample sizes, and limited follow-up data. High risk of bias was common across studies, including lack of randomisation concealment, blinding, and selective outcome reporting. Short follow-up periods further limit confidence in the durability of observed effects. Given the very high heterogeneity observed for key outcomes, pooled effect sizes are likely to vary according to intervention characteristics, baseline levels of distress, and the measurement instruments used. In addition, although this review was designed to evaluate psychological interventions for parents of children eligible for PPC, the available evidence was almost exclusively derived from paediatric oncology populations. Parents of children with cancer represent only a subset of the broader PPC population, as in many PPC services non-oncological conditions account for a substantial proportion of referrals, potentially limiting the generalizability of findings focused primarily on oncology populations (Maffeo et al., 2024). Consequently, the current evidence base may not adequately reflect the diversity and complexity of families typically served in PPC services, therefore, the findings of this review should be interpreted as providing indirect evidence relevant to PPC. Furthermore, we were unable to determine the severity of the cancer in the children included in the studies, making it difficult to understand which subpopulation might benefit the most and which ones might have been receiving PPC.

This highlights the urgent need to investigate psychological interventions for parents of children with non-oncological life-limiting or life-threatening conditions, such as neurological, metabolic, or genetic disorders, which are also relevant to PPC. Future research should address intervention heterogeneity by focusing on more homogeneous samples, clearly describing delivery modes, dosage, theoretical frameworks, and outcome measures. Long-term follow-ups with intermediate assessments, attrition management strategies, and cost-effectiveness evaluations are essential. Future trials should explicitly report PPC involvement (team composition, timing relative to diagnosis, concurrent disease-directed treatment) to enable transferability.

### 4.3 Conceptual alignment with the scope of paediatric palliative care

Only one study was conducted explicitly in the context of PPC, specifically among children with advanced cancer. The remaining studies were conducted in broader paediatric oncology settings and did not explicitly frame the care context as palliative, highlighting a gap in the literature and limiting generalizability to PPC populations. This limited representation reflects a conceptual gap between existing research and the WHO definition of PPC, which promotes an early, holistic, and continuous approach starting at the diagnosis of a life-limiting or life-threatening condition (World Health Organization, 2018).

According to this definition, PPC is not restricted to end-of-life situations, but also includes children living with serious, progressive, or incurable illnesses that threaten or limit their lives, even when their prognosis is uncertain, and their life expectancy may be relatively longer (Association for Children with Life-Threatening or Terminal Conditions and Their Families (ACT), 2009; World Health Organization, 2018). From this perspective, PPC should begin at diagnosis and can be delivered concurrently with disease-directed treatments, ensuring ongoing psychological and emotional support for children and their families (Thompson et al., 2024).

Therefore, the findings of this review should be interpreted as providing indirect evidence relevant to PPC, primarily reflecting parents of children with cancer who may be eligible for palliative care at different stages. Future research should explicitly adopt this broader conceptualization to evaluate psychological interventions across the full PPC continuum including non-terminal stages and earlier stages of illness where parental distress and adaptation processes are most dynamic.

### 4.4 Gender perspective and family diversity

An analysis was conducted to examine whether there were differences in outcomes between studies that included both parents and those that focused solely on mothers. This was motivated because, overall, the effects of interventions were rarely evaluated separately for fathers and mothers in the included studies, which limits understanding of potential variations in individual benefits according to sex, gender, and/or parental role.

Mothers typically show greater anxiety than parents both at diagnosis and throughout the disease trajectory, commonly because the primary caregiver role is generally attributed to mothers (Clarke et al., 2009; Kohlsdorf and Costa Junior, 2012). The results of this systematic review suggest that psychological and psychoeducational support interventions have been delivered either to mothers alone or to both parents. Across studies, no statistically significant subgroup differences were detected between interventions targeting mothers only and those involving both parents for outcomes including anxiety, depression, post-traumatic stress, and resilience. Involving both parents may be feasible, although evidence regarding differential effectiveness remains limited. In contexts where only mothers participate, such as in single-parent families, interventions may still produce meaningful effects, although evidence regarding comparative effectiveness with both parents is limited. Future research should explore whether mothers and fathers participating in the same study benefit similarly from psychological support, and how intervention design can accommodate families with non-traditional caregiving roles or diverse sociocultural backgrounds. Addressing gender and family diversity in future research is essential to ensure that psychological interventions in PPC are equitable, inclusive, and responsive to different caregiving configurations.

### 4.5 Clinical implications and implementation considerations

The integration of psychological care into the routine care of children with any life-threatening illness should be viewed as a core component and not limited to severe contexts, including advanced cancer or palliative care units. Implementing brief, flexible, and family-centred psychological programs within PPC services could help to alleviate emotional distress and enhancing parental coping during one of the most challenging phases of caregiving.

Embedding psychologists within interdisciplinary teams enables continuous screening, early intervention, and the prevention of cumulative psychological burden on family members. Sustainable practice also requires expanding training in paediatric palliative psychology and implementing institutional policies that formally integrate psychological care into PPC (Thompson et al., 2023, 2024). Future interventions should be theory-driven, with consistent structure, duration, and delivery format, and delivered by qualified psychologists experienced in PPC or the child’s underlying condition. Incorporating psychologists as core team members, rather than external consultants, ensures continuity and coherence in family-centred psychological support.

### 4.6 Timing and targets of psychological interventions

The differential pattern of effects observed across outcomes highlights the importance of considering both the timing and the specific targets of psychological interventions for parents. It is also important to note that heterogeneity in intervention characteristics, including type, duration, delivery mode, and outcome measures, may have influenced the observed differential effects across outcomes. Some interventions targeted acute emotional distress with brief formats, whereas others addressed broader adaptation processes through longer or multi-session programs, contributing to variable responsiveness across psychological and quality-of-life outcomes. This variability underscores that effect sizes reported across studies may not be directly comparable, and conclusions regarding intervention effectiveness should be interpreted cautiously. Considering this heterogeneity is essential when designing future interventions and interpreting effect patterns.

Outcomes related to acute emotional distress, such as anxiety, depressive symptoms, psychological distress, and hope, appeared to be more responsive to brief interventions, particularly in the short term. In contrast, broader or more stable dimensions of adjustment, including general psychological well-being or coping styles, showed more heterogeneous or non-significant effects.

This distinction suggests that psychological interventions in PPC may benefit from a stepped or phased approach, in which early interventions focus on alleviating acute distress, followed by more sustained or tailored support to address longer-term adaptation processes. Aligning intervention targets with parents’ evolving needs across the illness trajectory may enhance both effectiveness and clinical relevance.

### 4.7 Limitations

This SR and MA have several limitations that should be considered when interpreting the findings. Most notably, there was substantial methodological heterogeneity across the included studies, including variability in intervention type, duration, delivery format, and outcome measures. This heterogeneity, together with construct overlap and the frequent assessment of multiple outcomes, raises concerns about selective outcome reporting and multiplicity, and may have influenced the pooled effect estimates.

Studies quality limits the strength of the conclusions. Most trials were small and of low methodological quality, often lacking randomisation concealment, blind assessment, or sufficient statistical power. In addition, follow-up data were scarce, which precluded robust conclusions regarding the long-term sustainability of psychological benefits. Reporting bias cannot be excluded, since studies with non-significant results may be underrepresented. Adverse events were rarely assessed or reported, limiting conclusions about safety.

At the population level, the generalizability of the findings is restricted. Although this review focused on PPC, the evidence base identified was almost exclusively limited to paediatric oncology, which restricts the generalizability of the findings to other PPC populations, particularly those with non-oncological conditions. Additionally, most studies were conducted in high-income countries, and few reported or stratified outcomes by parental gender or family structure.

Finally, outcome assessment relied on a wide range of self-reported measures, many of which were not specifically developed or validated for parents of children in PPC contexts, potentially limiting sensitivity to change and cross-study comparability. At the review level, the exclusion of grey literature and the small number of comparable studies precluded subgroup meta-analyses.

Despite these limitations, this SR provides valuable evidence supporting the potential short-term benefits of psychological interventions for parents in the broader context of PPC and underscores the need for more rigorous, theory-driven studies with improved methodological quality, longer follow-up, and broader inclusion of non-oncological PPC populations.

## 5 Conclusion

In summary, this SR highlights that psychological interventions for caregivers or parents of children with cancer can produce meaningful short-term improvements in anxiety, depression, psychological distress, and hope, as well as notable long-term reductions in post-traumatic stress. However, the evidence is largely derived from paediatric oncology populations and only one study was PPC-specific, limiting generalizability. Psychological interventions in paediatric oncology settings appear to reduce parental anxiety and depression in the short term and may confer longer-term benefits for post-traumatic stress symptoms, but the evidence is heterogeneous and largely not drawn from PPC settings. Nonetheless, despite these encouraging findings, the overall quality of evidence remains limited due to methodological variability, small sample sizes, high risk of bias and insufficient follow-up data.

Consequently, advancing research and clinical practice in this field requires dual commitment: methodological rigor and compassionate, family-centred care. Notably, the predominance of oncology populations and the limited involvement of psychologists in intervention delivery highlight a gap between empirical research and recommended models of comprehensive, interdisciplinary PPC. Psychological support for parents and families in PPC should be recognized not as optional, but as an essential component of quality PPC. Robust PPC-embedded trials across non-oncological conditions and culturally diverse populations are urgently needed to investigate psychological interventions for parents of children with non-oncological life-limiting or life-threatening conditions, to better understand their effectiveness and inform evidence-based clinical practice.

## Data Availability

The datasets presented in this study can be found in online repositories. The names of the repository/repositories and accession number(s) can be found in the article/Supplementary material.

## Footnotes

1
https://www.sign.ac.uk/using-our-guidelines/methodology/search-filters/

## Supplementary material

SUPPLEMENTARY FIGURE S1 Summary risk of bias.

SUPPLEMENTARY FIGURE S2 Anxiety: Egger’s test.

SUPPLEMENTARY FIGURE S3 Anxiety: subgroup analysis by population.

SUPPLEMENTARY FIGURE S4 Depression: Egger’s test.

SUPPLEMENTARY FIGURE S5 Depression: subgroup analysis by population.

SUPPLEMENTARY FIGURE S6 Health-related quality of life: subgroup analysis by population.

SUPPLEMENTARY FIGURE S7 Posttraumatic stress symptoms: subgroup analysis by population.

SUPPLEMENTARY FIGURE S8 Stress: subgroup analysis by population.

SUPPLEMENTARY FIGURE S9 Resilience: subgroup analysis by population.
