# Supplementary figures and images for: Effectiveness of psychological interventions for parents of children eligible for paediatric palliative care: a systematic review and meta-analysis
Source: Front Psychol. 2026 Mar 3;17:1775937. doi: 10.3389/fpsyg.2026.1775937 (PMC12992277; doi:10.3389/fpsyg.2026.1775937)

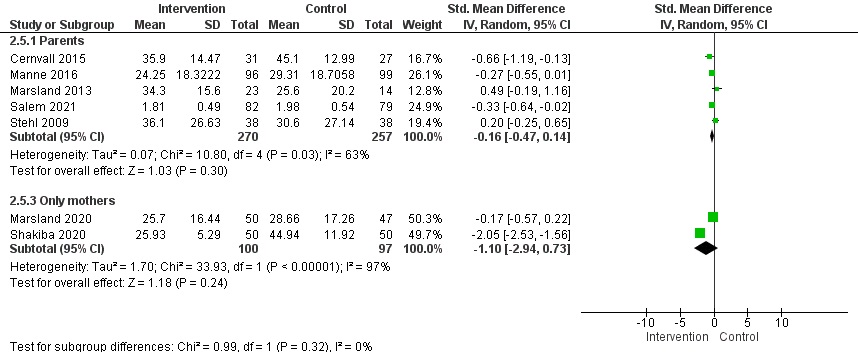

Supplement: SUPPLEMENTARY FIGURE S1 — Summary risk of bias. [file Image_1.JPEG]

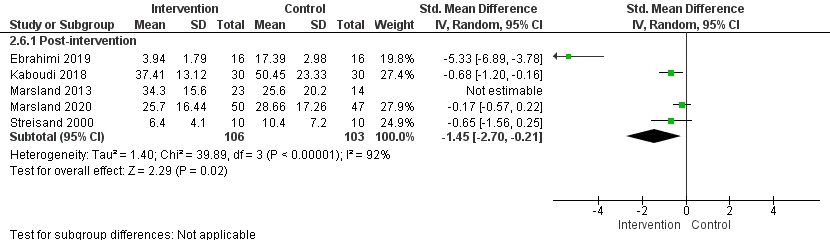

Supplement: SUPPLEMENTARY FIGURE S2 — Anxiety: Egger’s test. [file Image_2.JPEG]

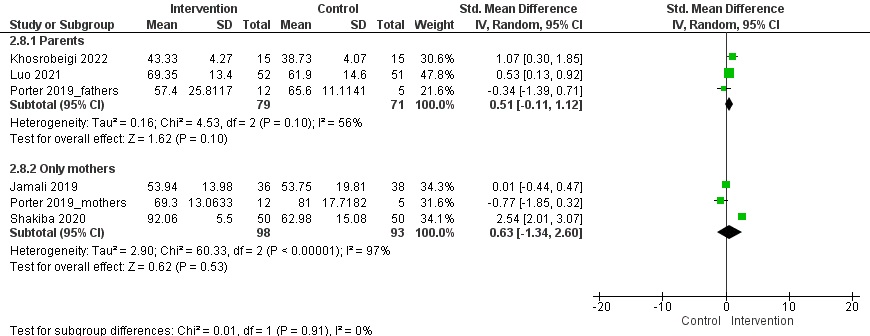

Supplement: SUPPLEMENTARY FIGURE S3 — Anxiety: subgroup analysis by population. [file Image_3.JPEG]

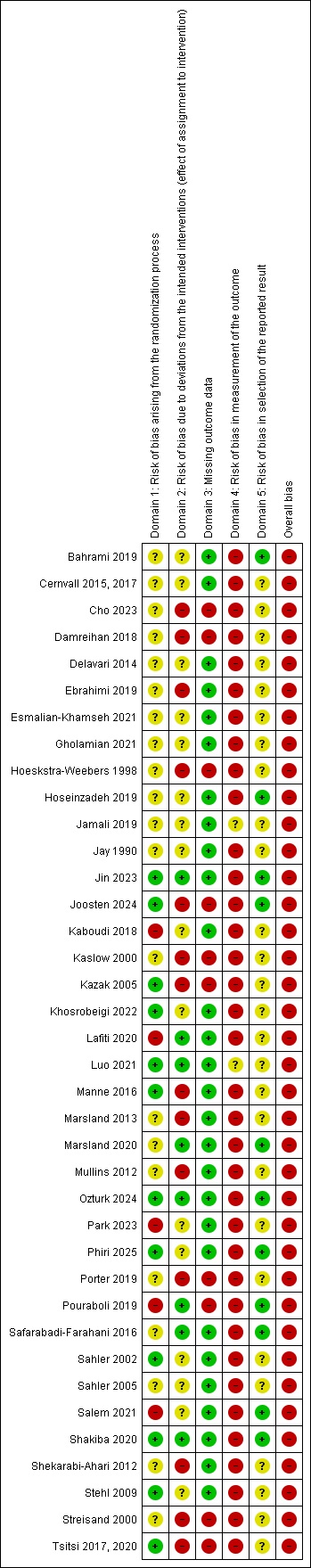

Supplement: SUPPLEMENTARY FIGURE S4 — Depression: Egger’s test. [file Image_4.JPEG]

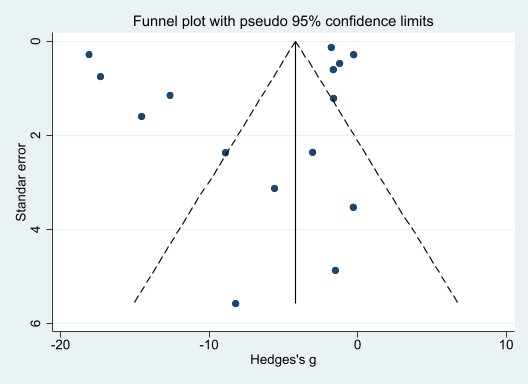

Supplement: SUPPLEMENTARY FIGURE S5 — Depression: subgroup analysis by population. [file Image_5.JPEG]

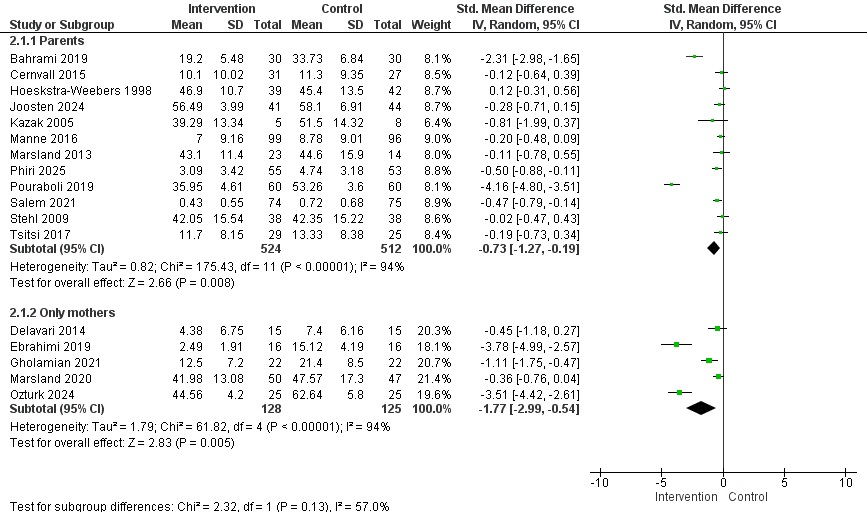

Supplement: SUPPLEMENTARY FIGURE S6 — Health-related quality of life: subgroup analysis by population. [file Image_6.JPEG]

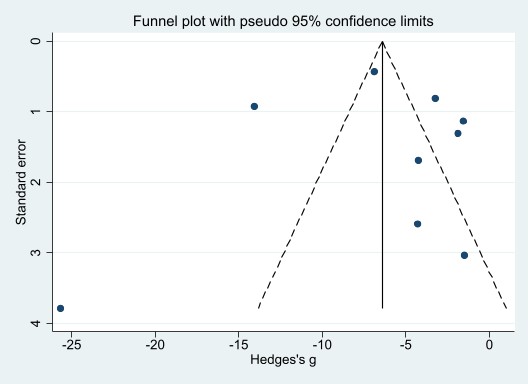

Supplement: SUPPLEMENTARY FIGURE S7 — Posttraumatic stress symptoms: subgroup analysis by population. [file Image_7.JPEG]

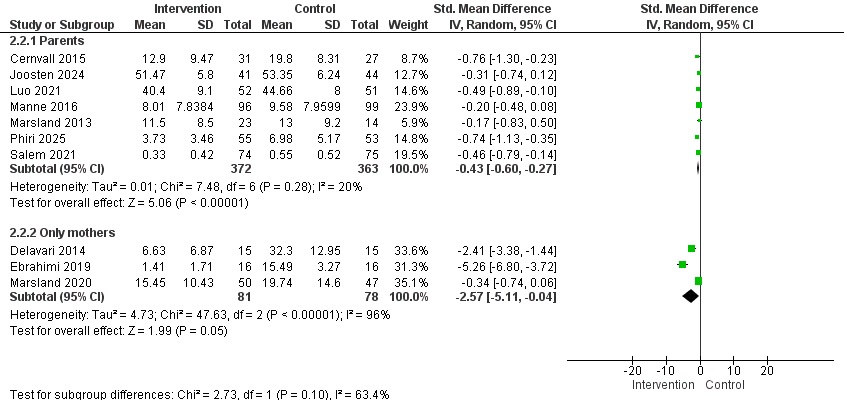

Supplement: SUPPLEMENTARY FIGURE S8 — Stress: subgroup analysis by population. [file Image_8.JPEG]

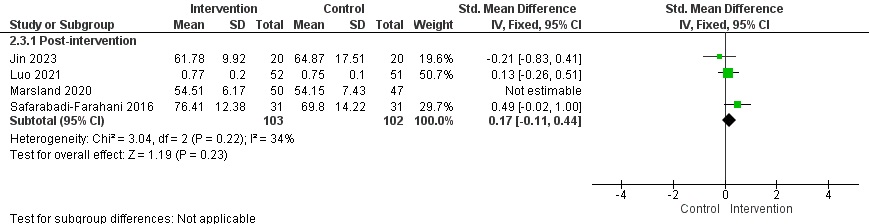

Supplement: SUPPLEMENTARY FIGURE S9 — Resilience: subgroup analysis by population. [file Image_9.JPEG]
