# Supplementary material for: Effectiveness of psychological interventions for parents of children eligible for paediatric palliative care: a systematic review and meta-analysis
Source: Front Psychol. 2026 Mar 3;17:1775937. doi: 10.3389/fpsyg.2026.1775937 (PMC12992277; doi:10.3389/fpsyg.2026.1775937)
Supplement: Supplementary file 11 [file Table_2.DOCX]

**Supplementary File, Table 2. Detailed search strategies**

| **ACCESS DATE:** 4th July 2024  **DATABASE:** MEDLINE(R) ALL <1946 to June 25, 2024>  **PLATFORM:** Ovid  **SEARCH FILTER:**  Randomized Clinical Trial - In: Scottish Intercollegiate Guidelines Network https://www.sign.ac.uk/using-our-guidelines/methodology/search-filters/ Accessed 2024-07-04 | | |
| --- | --- | --- |
| **#** | **Search** | **Results** |
| 1 | Psychotherapy/mt or "Psychotherapy, Brief"/ or Professional-Family Relations/ or Adaptation, Psychological/ or *Stress, Psychological/th or Family Therapy/ | 147321 |
| 2 | (psychotherap* or psychoeducat* or psychooncolog*).ti,ab,kf,kw. | 63670 |
| 3 | ((psychological or psychosocial or famil* or "stress management") adj (therap* or treatment* or intervention*)).ti,ab,kf,kw. | 37543 |
| 4 | *Mental Health/ | 35047 |
| 5 | Cognitive Behavioral Therapy/ or Behavior Therapy/mt | 43038 |
| 6 | ((cognitive* or "cognitive behavioral" or cognitive-existential or existential or dignity) adj therap*).ti,ab,kf,kw. | 4967 |
| 7 | Acceptance and Commitment Therapy/ | 1073 |
| 8 | ((dignity or interpersonal or "acceptance and Commitment") adj therap*).ti,ab,kf,kw. | 2655 |
| 9 | Relaxation Therapy/ | 6617 |
| 10 | ("life review" or "meaning making" or "emotional regulation" or mindfulness or coping or conversation* or "group discussion").ti,ab,kf,kw. | 131698 |
| 11 | *Problem Solving/ | 11614 |
| 12 | (problem solving or problem-solving).ti,ab,kf,kw. | 23399 |
| 13 | Social Support/ | 80682 |
| 14 | (support* adj (therapy or intervention*)).ti,ab,kw. | 12151 |
| 15 | *Advance Care Planning/ | 3576 |
| 16 | Advance Care Planning.ti,ab,kf,kw. | 5435 |
| 17 | or/1-16 | 481688 |
| 18 | exp infants/ or "Infant, Newborn"/ or exp Child/ or "Child, Preschool"/ or Pediatrics/ or exp adolescent/ or minors/ | 4083490 |
| 19 | (infant* or infants or infancy or newborn* or newborns or "new-born*" or perinat* or neonat* or baby* or babies or toddler* or toddlersor boys or boyhood or kids or child or child-related or childhood* or children* or adolescent* or adolescents or adolescence* or juvenile* or youth* or teen or teens* or pubescent* or pubescence or pediatric or pediatrics or paediatric or paediatrics or peadiatric or peadiatrics).ti,ab,kf,kw. | 2928596 |
| 20 | 18 or 19 | 4967879 |
| 21 | *Parents/px or Parenting/ or *Parent-Child Relations/ or fathers/px or mothers/px or Single parent/px or Family/ or *Legal Guardians/ or Caregivers/px | 180519 |
| 22 | (parent or parents or mother* or mothers or father* or fathers or caregiver* or caregivers or "care-giver" or "care-givers" or carers* or carer* or caring or caregiving or relatives* or legal guardia* or families* or family therapy or family-based therapy or family member*).ti,ab,kf,kw. | 1109634 |
| 23 | 21 or 22 | 1164690 |
| 24 | *Palliative Care/px or *Hospice Care/px or *Precursor Cell Lymphoblastic Leukemia-Lymphoma/px or Death/ or *Leukemia/px or Neoplasms/px or *Stem Cell Transplantation/px or Hematopoietic Stem Cell Transplantation/px or Terminally Ill/ | 52905 |
| 25 | (palliat* or ((terminal* or catastroph* or death) adj2 (disease* or care or ill or illness)) or hospice or hospices or (end adj2 life) or "end-of-life" or "medical complexit*" or "complex medical condition*" or "complex chronic condition*" or "multiple complex chronic" or "medical fragility" or "life-threatening" or "life-limiting" or "long term hospitalization" or dying).ti,ab,kf,kw. | 302539 |
| 26 | Transplantation/px or End Stage Liver Disease/px or Cystic Fibrosis/px or "Muscular Dystrophy, Duchenne"/px or Epidermolysis Bullosa/px or Neuromuscular Diseases/px or Neurodegenerative Diseases/px or Cerebral Palsy/px | 3902 |
| 27 | ("transplantation" or "end-stage organ failure" or "cystic fibrosis" or "Duchenne Muscular Dystrophy" or "Epidermolysis Bullosa" or "Neuromuscular Disorder*" or "Neurodegenerative Disorder*" or "Progressive Metabolic Disorder*" or "Advanced Cancer" or "invasive cancer" or Metastas* or ((child* or p?ediatric) adj2 (leukemia* or leukaemia* or cancer* or malignan* or myeloma or lymphoma or meningioma or seriously-ill)) or "Severe Cerebral Palsy" or "Multimorphic Syndromes").ti,ab,kf,kw. | 1074859 |
| 28 | Bereavement/ or *Grief/ | 11864 |
| 29 | (bereaved or bereavement or grief or grieving or mourning or cancer-bereave* or Pre-loss or deathbed or death-bed).ti,ab,kf,kw. | 18936 |
| 30 | ((recent or before or after or traumati* or child* or cancer or loved) adj (loss or death)).ti,ab,kf,kw. | 26517 |
| 31 | 24 or 25 or 26 or 27 or 28 or 29 or 30 | 1415559 |
| 32 | 17 and 20 and 23 and 31 | 6836 |
| 33 | Randomized Controlled Trials as Topic/ | 171387 |
| 34 | randomized controlled trial/ | 616285 |
| 35 | Random Allocation/ | 107388 |
| 36 | Double Blind Method/ | 179245 |
| 37 | Single Blind Method/ | 33665 |
| 38 | clinical trial/ | 540141 |
| 39 | clinical trial, phase i.pt. | 26116 |
| 40 | clinical trial, phase ii.pt. | 41546 |
| 41 | clinical trial, phase iii.pt. | 22947 |
| 42 | clinical trial, phase iv.pt. | 2512 |
| 43 | controlled clinical trial.pt. | 95565 |
| 44 | randomized controlled trial.pt. | 616285 |
| 45 | multicenter study.pt. | 349429 |
| 46 | clinical trial.pt. | 540141 |
| 47 | exp Clinical Trials as topic/ | 394010 |
| 48 | or/33-47 | 1614974 |
| 49 | (clinical adj trial$).tw. | 517107 |
| 50 | ((singl$ or doubl$ or treb$ or tripl$) adj (blind$3 or mask$3)).tw. | 206196 |
| 51 | PLACEBOS/ | 35967 |
| 52 | placebo$.tw. | 257663 |
| 53 | randomly allocated.tw. | 38972 |
| 54 | (allocated adj2 random$).tw. | 42913 |
| 55 | or/49-54 | 833130 |
| 56 | 48 or 55 | 1993784 |
| 57 | case report.tw. | 430733 |
| 58 | letter/ | 1261009 |
| 59 | historical article/ | 370581 |
| 60 | or/57-59 | 2041978 |
| 61 | 56 not 60 | 1949599 |
| 62 | 32 and 61 | 522 |
| **ACCESS DATE:** 4th July 2024  **DATABASE:** Excerpta Medica dataBASE (Embase)  **PLATFORM:** Elsevier  **SEARCH FILTER:**  Randomized Clinical Trial - In: Scottish Intercollegiate Guidelines Network <https://www.sign.ac.uk/using-our-guidelines/methodology/search-filters/> Accessed 2024-07-04 | | |
| **No.** | **Query** | **Results** |
| #1 | 'psychotherapy'/exp/dm_th OR 'short term psychotherapy'/de OR 'psychological adjustment'/de OR 'mental stress'/mj | 42820 |
| #2 | psychotherap*:ti,ab,kw OR psychoeducat*:ti,ab,kw OR psychooncolog*:ti,ab,kw | 97240 |
| #3 | ((psychological OR psychosocial OR famil* OR 'stress management') NEXT/1 (therap* OR treatment* OR intervention*)):ti,ab,kw | 47015 |
| #4 | 'mental health'/mj | 69164 |
| #5 | 'cognitive behavioral therapy'/de OR 'behavior therapy'/de | 76341 |
| #6 | ((cognitive* OR 'cognitive behavioral' OR 'cognitive existential' OR existential OR dignity) NEXT/1 therap*):ti,ab,kw | 26862 |
| #7 | 'acceptance and commitment therapy'/de | 3007 |
| #8 | ((dignity OR interpersonal OR 'acceptance and commitment') NEXT/1 therap*):ti,ab,kw | 3536 |
| #9 | 'relaxation training'/de | 12538 |
| #10 | 'life review':ti,ab,kw OR 'meaning-making':ti,ab,kw OR 'emotion regulation':ti,ab,kw OR 'mindfulness':ti,ab,kw OR 'coping':ti,ab,kw OR 'conversation*':ti,ab,kw OR 'group discussion':ti,ab,kw | 182439 |
| #11 | 'problem solving'/mj | 10595 |
| #12 | 'problem solving':ti,ab,kw | 28972 |
| #13 | 'social support'/de | 121627 |
| #14 | 'support intervention*':ti,ab,kw | 4766 |
| #15 | 'advance care planning'/mj | 3679 |
| #16 | 'advance care planning':ti,ab,kw | 8950 |
| #17 | #1 OR #2 OR #3 OR #4 OR #5 OR #6 OR #7 OR #8 OR #9 OR #10 OR #11 OR #12 OR #13 OR #14 OR #15 OR #16 | 617213 |
| #18 | 'infant'/exp OR 'newborn'/de OR 'child'/exp OR 'preschool child'/de OR 'pediatric'/de OR 'adolescent'/exp OR 'newborn disease'/exp OR 'minor (person)'/de | 5874216 |
| #19 | infant*:ti,ab,kw OR infants:ti,ab,kw OR infancy:ti,ab,kw OR newborn*:ti,ab,kw OR newborns:ti,ab,kw OR 'new born*':ti,ab,kw OR perinat*:ti,ab,kw OR neonat*:ti,ab,kw OR baby*:ti,ab,kw OR babies:ti,ab,kw OR toddler*:ti,ab,kw OR toddlers:ti,ab,kw OR minor:ti,ab,kw OR minors:ti,ab,kw OR boys:ti,ab,kw OR boyhood:ti,ab,kw OR kids:ti,ab,kw OR child:ti,ab,kw OR 'child related':ti,ab,kw OR childhood*:ti,ab,kw OR children*:ti,ab,kw OR adolescent*:ti,ab,kw OR adolescents:ti,ab,kw OR adolescence*:ti,ab,kw OR juvenile*:ti,ab,kw OR youth*:ti,ab,kw OR teen:ti,ab,kw OR teens*:ti,ab,kw OR pubescent*:ti,ab,kw OR pubescence:ti,ab,kw OR pediatric:ti,ab,kw OR pediatrics:ti,ab,kw OR paediatric:ti,ab,kw OR paediatrics:ti,ab,kw OR peadiatric:ti,ab,kw OR peadiatrics:ti,ab,kw | 4141911 |
| #20 | #18 OR #19 | 7106924 |
| #21 | 'parent'/mj OR 'child parent relation'/mj OR 'father'/de OR 'mother'/de OR 'single parent'/de OR 'family'/de OR 'family therapy'/de OR 'legal guardian'/mj OR 'caregiver'/de | 404402 |
| #22 | parent:ti,ab,kw OR parents:ti,ab,kw OR mother*:ti,ab,kw OR mothers:ti,ab,kw OR father*:ti,ab,kw OR fathers:ti,ab,kw OR family*:ti,ab,kw OR families*:ti,ab,kw OR caregiver*:ti,ab,kw OR caregivers:ti,ab,kw OR 'care giver':ti,ab,kw OR 'care givers':ti,ab,kw OR carers*:ti,ab,kw OR carer*:ti,ab,kw OR caring:ti,ab,kw OR caregiving:ti,ab,kw OR relatives*:ti,ab,kw OR 'legal guardia*':ti,ab,kw | 2310979 |
| #23 | #21 OR #22 | 2376823 |
| #24 | 'palliative therapy'/mj OR 'hospice care'/mj OR 'acute lymphoid leukemia cell line'/mj OR 'death'/de OR 'leukemia'/mj OR 'neoplasm'/de OR 'stem cell transplantation'/mj OR 'hematopoietic stem cell transplantation'/de OR 'terminally ill patient'/de | 1029560 |
| #25 | palliat*:ti,ab,kw OR (((terminal* OR catastroph* OR death) NEAR/2 (disease* OR care OR ill OR illness)):ti,ab,kw) OR hospice:ti,ab,kw OR hospices:ti,ab,kw OR ((end NEAR/2 life):ti,ab,kw) OR 'end of life':ti,ab,kw OR 'medical complexit*':ti,ab,kw OR 'complex medical condition*':ti,ab,kw OR 'complex chronic condition*':ti,ab,kw OR 'multiple complex chronic':ti,ab,kw OR 'medical fragility':ti,ab,kw OR 'life threatening':ti,ab,kw OR 'life limiting':ti,ab,kw OR 'long term hospitalization':ti,ab,kw OR dying:ti,ab,kw | 450332 |
| #26 | 'transplantation'/de OR 'end stage liver disease'/de OR 'cystic fibrosis'/de OR 'duchenne muscular dystrophy'/de OR 'epidermolysis bullosa'/de OR 'neuromuscular disease'/de OR 'degenerative disease'/de OR 'cerebral palsy'/de | 453660 |
| #27 | transplantation:ti,ab,kw OR 'end-stage organ failure':ti,ab,kw OR 'cystic fibrosis':ti,ab,kw OR 'duchenne muscular dystrophy':ti,ab,kw OR 'epidermolysis bullosa':ti,ab,kw OR 'neuromuscular disorder*':ti,ab,kw OR 'neurodegenerative disorder*':ti,ab,kw OR 'progressive metabolic disorder*':ti,ab,kw OR 'advanced cancer':ti,ab,kw OR 'invasive cancer':ti,ab,kw OR metastas*:ti,ab,kw OR (((child* OR p$ediatric) NEAR/2 (leukemia* OR leukaemia* OR cancer* OR malignan* OR myeloma OR lymphoma OR meningioma OR 'seriously ill')):ti,ab,kw) OR 'severe cerebral palsy':ti,ab,kw OR 'multimorphic syndromes':ti,ab,kw | 1597640 |
| #28 | 'bereavement'/de | 11855 |
| #29 | bereaved:ti,ab,kw OR bereavement:ti,ab,kw OR grief:ti,ab,kw OR grieving:ti,ab,kw OR mourning:ti,ab,kw OR 'cancer bereave*':ti,ab,kw OR 'pre loss':ti,ab,kw OR deathbed:ti,ab,kw OR 'death bed':ti,ab,kw | 25402 |
| #30 | ((recent OR before OR after OR traumati* OR child* OR cancer OR loved) NEXT/1 (loss OR death)):ti,ab,kw | 37327 |
| #31 | #24 OR #25 OR #26 OR #27 OR #28 OR #29 OR #30 | 3041556 |
| #32 | #17 AND #20 AND #23 AND #31 | 9186 |
| #33 | #32 AND [embase]/lim | 6630 |
| #34 | 'clinical trial'/de | 899691 |
| #35 | 'randomized controlled trial'/de | 430335 |
| #36 | 'controlled clinical trial'/de | 388700 |
| #37 | 'multicenter study'/de | 145218 |
| #38 | 'phase 3 clinical trial'/de | 25912 |
| #39 | 'phase 4 clinical trial'/de | 2321 |
| #40 | 'randomization'/exp | 72066 |
| #41 | 'single blind procedure'/de | 25319 |
| #42 | 'double blind procedure'/de | 134651 |
| #43 | 'crossover procedure'/de | 49392 |
| #44 | 'placebo'/de | 300200 |
| #45 | 'randomi*ed controlled trial*':ti,ab | 150973 |
| #46 | rct:ti,ab | 22807 |
| #47 | (random* NEAR/2 allocat*):ti,ab | 32419 |
| #48 | 'single blind*':ti,ab | 18918 |
| #49 | 'double blind*':ti,ab | 175660 |
| #50 | ((treble OR triple) NEAR/1 blind*):ti,ab | 725 |
| #51 | placebo*:ti,ab | 247561 |
| #52 | 'prospective study'/de | 353547 |
| #53 | #34 OR #35 OR #36 OR #37 OR #38 OR #39 OR #40 OR #41 OR #42 OR #43 OR #44 OR #45 OR #46 OR #47 OR #48 OR #49 OR #50 OR #51 OR #52 | 1747732 |
| #54 | 'case study'/de | 44113 |
| #55 | 'case report':ti,ab | 331502 |
| #56 | 'abstract report'/de OR 'letter'/de | 1001369 |
| #57 | 'conference paper'/it | 741694 |
| #58 | 'conference abstract'/it | 2447768 |
| #59 | 'conference proceeding'/it | 0 |
| #60 | 'editorial'/it | 519433 |
| #61 | 'letter'/it | 955398 |
| #62 | 'note'/it | 656399 |
| #63 | #54 OR #55 OR #56 OR #57 OR #58 OR #59 OR #60 OR #61 OR #62 | 5705375 |
| #64 | #53 NOT #63 | 2147863 |
| #65 | #33 AND #64 | 360 |
| **ACCESS DATE:** 4th July 2024  **DATABASE:** Cumulative Index to Nursing and Allied Health Literature (CINAHL)  **PLATFORM:** EBSCOhost  **SEARCH FILTER:**  Randomized Clinical Trial - In: Scottish Intercollegiate Guidelines Network https://www.sign.ac.uk/using-our-guidelines/methodology/search-filters/ Accessed 2024-07-04 | | |
| **#** | **Query** | **Results** |
| S1 | (MH "Psychotherapy/MT") OR (MH "Psychotherapy, Brief") OR (MH "Professional-Family Relations") OR (MH "Adaptation, Psychological") OR (MM "Stress, Psychological"/TH) OR MH "Family therapy") | 68,629 |
| S2 | ((TI psychotherap* OR AB psychotherap*) OR (TI psychoeducat* OR AB psychoeducat*) OR (TI psychooncolog* OR AB psychooncolog*)) | 21,61 |
| S3 | (((TI psychological OR AB psychological) OR (TI psychosocial OR AB psychosocial) OR (TI famil* OR AB famil*) OR (TI "stress management" OR AB "stress management")) N0 ((TI therap* OR AB therap*) OR (TI treatment* OR AB treatment) OR (TI intervention* OR AB intervention*))) | 18,517 |
| S4 | (MM "Mental Health"/TH) | 9,117 |
| S5 | (MH "Cognitive Behavioral Therapy") OR (MH "Behavior Therapy"/MT) | 1,123 |
| S6 | (((TI cognitive* OR AB cognitive*) OR (TI "cognitive behavioral" OR AB "cognitive behavioral") OR (TI cognitive-existential OR AB cognitive-existential) OR (TI existential OR AB existential) OR (TI dignity OR AB dignity)) N0 (TI therap* OR AB therap*)) | 8,839 |
| S7 | (MH "Acceptance and Commitment Therapy") | 1,058 |
| S8 | (((TI dignity OR AB dignity) OR (TI interpersonal OR AB interpersonal) OR (TI "acceptance and Commitment" OR AB "acceptance and Commitment")) N0 (TI therap* OR AB therap*)) | 1,664 |
| S9 | (MM "Simple Relaxation Therapy" | 8,349 |
| S10 | ((TI "life review" OR AB "life review") OR (TI "meaning making" OR AB "meaning making") OR (TI "emotional regulation" OR AB "emotional regulation") OR (TI mindfulness OR AB mindfulness) OR (TI coping OR AB coping) OR (TI conversation* OR AB conversation*) OR (TI "group discussion" OR AB "group discussion")) | 80,872 |
| S11 | (MM "Problem Solving") | 5,136 |
| S12 | ((TI "problem solving" OR AB "problem solving") OR (TI problem-solving OR AB problem-solving) | 10,993 |
| S13 | (MM "Support, Social") | 2,417 |
| S14 | TI (support* N0 (therapy or intervention*)) OR AB (support* N0 (therapy or intervention*)) | 6,392 |
| S15 | (MM "Advance Care Planning") | 3,479 |
| S16 | (TI "Advance Care Planning" OR AB "Advance Care Planning") | 3,833 |
| S17 | S1 OR S2 OR S3 OR S4 OR S5 OR S6 OR S7 OR S8 OR S9 OR S10 OR S11 OR S12 OR S13 OR S14 OR S15 OR S16 | 197,326 |
| S18 | (MH infants) OR (MH "Infant, Newborn") OR (MH Child) OR (MH "Child, Preschool") OR (MH Pediatrics) OR (MH adolescent) OR (MH minors) | 715,791 |
| S19 | ((TI infant* OR AB infant*) OR (TI infants OR AB infants) OR (TI infancy OR AB infancy) OR (TI newborn* OR AB newborn*) OR (TI newborns OR AB newborns OR (TI new-born* OR AB new-born*) OR (TI perinat* OR AB perinat*) OR (TI neonat* OR AB neonat*) OR (TI baby* OR AB baby*) OR (TI babies OR AB babies) OR (TI toddler* OR AB toddler*) OR (TI toddlers OR AB toddlers) OR (TI boys OR AB boys) OR (TI boyhood OR AB boyhood) OR (TI kids OR AB kids) OR (TI child OR AB child) OR (TI child-related OR AB child-related) OR (TI childhood* OR AB childhood*) OR (TI children* OR AB children*) OR (TI adolescent* OR AB adolescent*) OR (TI adolescents OR AB adolescents) OR (TI adolescence* OR AB adolescence*) OR (TI juvenile* OR AB juvenile*) OR (TI youth* OR AB youth*) OR (TI teen OR AB teen) OR (TI teens* OR AB teens*) OR (TI pubescent* OR AB pubescent*) OR (TI pubescence OR AB pubescence OR SU pubescence) OR (TI pediatric OR AB pediatric) OR (TI pediatrics OR AB pediatrics) OR (TI paediatric OR AB paediatric ) OR (TI paediatrics OR AB paediatrics) OR (TI peadiatric OR AB peadiatric) OR (TI peadiatrics OR AB peadiatrics)) NOT "adult child" | 998,828 |
| S20 | S18 OR S19 | 1,200,650 |
| S21 | (MH Parents/PX) OR (MM Parenting) OR (MM "Parent-Child Relations") OR (MH fathers/PX) OR (MH mothers/PX) OR (MH "Single parent"/PX) OR (MH Family) OR (MH "Family Therapy") OR (MM "Legal Guardians") OR (MH Caregivers/PX) | 79,48 |
| S22 | (TI parent OR AB parent) OR (TI parents OR AB parents) OR (TI mother* OR AB mother*) OR (TI mothers OR AB mothers) OR (TI father* OR AB father*) OR (TI fathers OR AB fathers) OR (TI caregiver* OR AB caregiver*) OR (TI caregivers OR AB caregivers) OR (TI care-giver OR AB care-giver) OR (TI care-givers OR AB care-givers) OR (TI carers* OR AB carers*) OR (TI carer* OR AB carer*) OR (TI caring OR AB caring) OR (TI caregiving OR AB caregiving) OR (TI relatives* OR AB relatives*) OR (TI "legal guardia*" OR AB "legal guardia*") OR (TI families* OR AB families*) OR (TI "family therapy" OR AB "family therapy") OR (TI "family based" OR AB "family-based") OR (TI "family member*" OR AB "family member*")) | 424,804 |
| S23 | S21 OR S22 | 456,086 |
| S24 | (MM "Palliative Care"/PX) OR (MM "Hospice Care"/PX) OR (MM "Precursor Cell Lymphoblastic Leukemia-Lymphoma"/PX) OR (MM Death/PX) OR (MM Leukemia/PX) OR (MM Neoplasms/PX) OR (MM "Stem Cell Transplantation"/PX) OR (MM "Hematopoietic Stem Cell Transplantation"/PX) OR (MM "Terminally Ill") | 257 |
| S25 | ((TI palliat* OR AB palliat*) OR (((TI terminal* OR AB terminal*) OR (TI catastroph* OR AB catastroph*) OR (TI death OR AB death)) N1 ((TI disease* OR AB disease*) OR (TI care OR AB care) OR (TI ill OR AB ill) OR (TI illness OR AB illness))) OR (TI hospice OR AB hospice) OR (TI hospices OR AB hospices) OR ((TI end OR AB end) N1 (TI life OR AB life)) OR (TI end-of-life OR AB end-of-life) OR (TI "medical complexit*" OR AB "medical complexit*") OR (TI "complex medical condition*" OR AB "complex medical condition*") OR (TI "complex chronic condition*" OR AB "complex chronic condition*") OR (TI "multiple complex chronic" OR AB "multiple complex chronic") OR (TI "medical fragility" OR AB "medical fragility") OR (TI life-threatening OR AB life-threatening) OR (TI life-limiting OR AB life-limiting) OR (TI "long term hospitalization" OR AB "long term hospitalization") OR (TI dying OR AB dying)) | 117,546 |
| S26 | (MH Transplantation/PX) OR (MH "End Stage Liver Disease"/PX) OR (MH "Cystic Fibrosis"/PX) OR (MH "Muscular Dystrophy, Duchenne"/PX) OR (MH "Epidermolysis Bullosa"/PX) OR (MH "Neuromuscular Diseases"/PX) OR (MH "Neurodegenerative Diseases"/PX) OR (MH "Cerebral Palsy"/PX) | 386 |
| S27 | ((TI transplantation OR AB transplantation) OR (TI "end-stage organ failure" OR AB "end-stage organ failure") OR (TI "cystic fibrosis" OR AB "cystic fibrosis") OR (TI "Duchenne Muscular Dystrophy" OR AB "Duchenne Muscular Dystrophy") OR (TI "Epidermolysis Bullosa" OR AB "Epidermolysis Bullosa") OR (TI "Neuromuscular Disorder*" OR AB "Neuromuscular Disorder*") OR (TI "Neurodegenerative Disorder*" OR AB "Neurodegenerative Disorder*") OR (TI "Progressive Metabolic Disorder*" OR AB "Progressive Metabolic Disorder*") OR (TI "Advanced Cancer" OR AB "Advanced Cancer") OR (TI "invasive cancer" OR AB "invasive cancer") OR (TI Metastas* OR AB Metastas*) OR (((TI child* OR AB child* OR SU child*) OR (TI p#ediatric OR AB p#ediatric)) N0 ((TI leukemia* OR AB leukemia*) OR (TI leukaemia* OR AB leukaemia*) OR (TI cancer* OR AB cancer*) OR (TI malignan* OR AB malignan*) OR (TI myeloma OR AB myeloma) OR (TI lymphoma OR AB lymphoma) OR (TI meningioma OR AB meningiomaa) OR (TI seriously-ill OR AB seriously-ill))) OR (TI "Severe Cerebral Palsy" OR AB "Severe Cerebral Palsy") OR (TI "Multimorphic Syndromes" OR AB "Multimorphic Syndromes") | 142,485 |
| S28 | (MH Bereavement) | 9,249 |
| S29 | ((TI bereaved OR AB bereaved) OR (TI bereavement OR AB bereavement) OR (TI grief OR AB grief) OR (TI grieving OR AB grieving) OR (TI mourning OR AB mourning) OR (TI cancer-bereave* OR AB cancer-bereave*) OR (TI Pre-loss OR AB Pre-loss) OR (TI deathbed OR AB deathbed) OR (TI death-bed OR AB death-bed)) | 15,645 |
| S30 | (((TI recent OR AB recent) OR (TI before OR AB before) OR (TI after OR AB after) OR (TI traumati* OR AB traumati*) OR (TI child* OR AB child*) OR (TI cancer OR AB cancer) OR (TI loved OR AB loved)) N0 ((TI loss OR AB loss) OR (TI death OR AB death))) | 16,096 |
| S31 | S24 OR S25 OR S26 OR S27 OR S28 OR S29 OR S30 | 278,531 |
| S32 | S17 AND S20 AND S23 AND S31 | 2,792 |
| S33 | (MH “Randomized Clinical Trials”) | 893 |
| S34 | PT Randomized Clinical Trials | 3,701 |
| S35 | TX clinic* N1 trial* | 476,334 |
| S36 | TX ((trebl* N1 blind*) OR (trebl* N1 mask*)) | 16 |
| S37 | TX ((tripl* N1 blind*) OR (tripl* N1 mask*)) | 1,478 |
| S38 | TX ((doubl* N1 blind*) OR (doubl* N1 mask*)) | 1,364,681 |
| S39 | TX ((singl* N1 blind*) OR (singl* N1 mask*)) | 27,688 |
| S40 | TX randomi* control* trial* | 390,274 |
| S41 | (MH “Random Assignment”) | 85,958 |
| S42 | TX random* allocat* | 27,214 |
| S43 | TX placebo* | 144,073 |
| S44 | (MH “Placebos”) | 14,484 |
| S45 | (MH “Quantitative Studies”) | 39,891 |
| S46 | TX allocat* random* | 4,909 |
| S47 | S33 OR S34 OR S35 OR S36 OR S37 OR S38 OR S39 OR S40 OR S41 OR S42 OR S43 OR S44 OR S45 OR S46 | 1,973,944 |
| S48 | S32 AND S47 | 938 |
| S49 | S32 AND S47 | 492 |
| S50 | S48 NOT S49 | 446 |
| **ACCESS DATE:** 4th July 2024  **DATABASE:** APA PsycINFO  **PLATFORM:** EBSCOhost  **SEARCH FILTER:** Adapted from Randomized Clinical Trial - In: Scottish Intercollegiate Guidelines Network https://www.sign.ac.uk/using-our-guidelines/methodology/search-filters/ Accessed 2024-07-04 | | |
| **#** | **Query** | **Results** |
| S1 | (MA "Psychotherapy") OR (MA "Brief Psychotherapy") | 32,597 |
| S2 | ((TI psychotherap* OR AB psychotherap* OR KW psychotherap*) OR (TI psychoeducat* OR AB psychoeducat* OR KW psychoeducat*) OR (TI psychooncolog* OR AB psychooncolog* OR KW psychooncolog*)) | 145,784 |
| S3 | (((TI psychological OR AB psychological OR KW psychological) OR (TI psychosocial OR AB psychosocial OR KW psychosocial) OR (TI famil* OR AB famil* OR KW famil*) OR (TI "stress management" OR AB "stress management" OR KW "stress management")) N0 ((TI therap* OR AB therap* OR KW therap*) OR (TI treatment* OR AB treatment* OR KW treatment*) OR (TI intervention* OR AB intervention* OR KW intervention*))) | 56,188 |
| S4 | MJ "mental health" AND MJ "therapy" | 5,264 |
| S5 | (MA "Cognitive Behavior Therapy") | 91,602 |
| S6 | (((TI cognitive* OR AB cognitive* OR KW cognitive*) OR (TI "cognitive behavioral" OR AB "cognitive behavioral" OR KW "cognitive behavioral") OR (TI cognitive-existential OR AB cognitive-existential OR KW cognitive-existential) OR (TI existential OR AB existential OR KW existential) OR (TI dignity OR AB dignity OR KW dignity)) N0 (TI therap* OR AB therap* OR KW therap*)) | 29,161 |
| S7 | (MJ "Acceptance and Commitment Therapy") | 2,529 |
| S8 | (((TI dignity OR AB dignity OR KW dignity) OR (TI interpersonal OR AB interpersonal OR KW interpersonal) OR (TI "acceptance and Commitment" OR AB "acceptance and Commitment" OR KW "acceptance and Commitment")) N0 (TI therap* OR AB therap* OR KW therap*)) | 4,658 |
| S9 | (MJ "Relaxation Therapy") | 3,213 |
| S10 | ((TI "life review" OR AB "life review") OR (TI "meaning making" OR AB "meaning making") OR (TI "emotional regulation" OR AB "emotional regulation") OR (TI mindfulness OR AB mindfulness) OR (TI coping OR AB coping) OR (TI conversation* OR AB conversation*) OR (TI "group discussion" OR AB "group discussion")) | 172,169 |
| S11 | (MJ "Problem Solving") | 26,685 |
| S12 | ((TI "problem solving" OR AB "problem solving" OR KW "problem solving") OR (TI problem-solving OR AB problem-solving) | 50,213 |
| S13 | (MJ "Social Support") | 34,785 |
| S14 | (TI "support intervention*" OR AB "support intervention*" OR KW "support intervention*") | 2,075 |
| S15 | (MJ "Advance Directives") | 1,912 |
| S16 | (TI "Advance Care Planning" OR AB "Advance Care Planning" OR KW "Advance Care Planning") | 1,83 |
| S17 | S1 OR S2 OR S3 OR S4 OR S5 OR S6 OR S7 OR S8 OR S9 OR S10 OR S11 OR S12 OR S13 OR S14 OR S15 OR S16 | 473,212 |
| S18 | ((TI infant* OR AB infant*) OR (TI infants OR AB infants OR (TI infancy OR AB infancy) OR (TI newborn* OR AB newborn*) OR (TI newborns OR AB newborns) OR (TI new-born* OR AB new-born*) OR (TI perinat* OR AB perinat) OR (TI neonat* OR AB neonat*) OR (TI baby* OR AB baby*) OR (TI babies OR AB babies) OR (TI toddler* OR AB toddler*) OR (TI toddlers OR AB toddlers) OR (TI minor OR AB minor) OR (TI minors OR AB minors) OR (TI boys OR AB boys) OR (TI boyhood OR AB boyhood) OR (TI kids OR AB kids) OR (TI child OR AB child) OR (TI child-related OR AB child-related) OR (TI childhood* OR AB childhood*) OR (TI children* OR AB children* OR KW children*) OR (TI adolescent* OR AB adolescent*) OR (TI adolescents OR AB adolescents) OR (TI adolescence* OR AB adolescence*) OR (TI juvenile* OR AB juvenile*) OR (TI youth* OR AB youth*) OR (TI teen OR AB teen) OR (TI teens* OR AB teens*) OR (TI pubescent* OR AB pubescent*) OR (TI pubescence OR AB pubescence) OR (TI pediatric OR AB pediatric) OR (TI pediatrics OR AB pediatrics) OR (TI paediatric OR AB paediatric) OR (TI paediatrics OR AB paediatrics) OR (TI peadiatric OR AB peadiatric) OR (TI peadiatrics OR AB peadiatrics)) | 1,159,992 |
| S19 | (MJ Parents) OR (MA fathers) OR (MA mothers) OR (MA "Single parents") OR (MA "Family Therapy") OR (MA Caregivers) | 113,455 |
| S20 | ((TI parent OR AB parent) OR (TI parents OR AB parents) OR (TI mother* OR AB mother*) OR (TI mothers OR AB mothers) OR (TI father* OR AB father*) OR (TI fathers OR AB fathers) OR (TI families* OR AB families*) OR (TI caregiver* OR AB caregiver*) OR (TI caregivers OR AB caregivers) OR (TI care-giver OR AB care-giver) OR (TI care-givers OR AB care-givers) OR (TI carers* OR AB carers*) OR (TI carer* OR AB carer*) OR (TI caring OR AB caring) OR (TI caregiving OR AB caregiving) OR (TI relatives* OR AB relatives*) OR (TI "legal guardia*" OR AB "legal guardia*")) | 559,99 |
| S21 | S19 OR S20 | 571,928 |
| S22 | (MJ "Palliative Care") OR (MJ "Hospice") OR (MJ "Child Death") OR (MJ "Terminally Ill patients") | 19,157 |
| S23 | ((TI palliat* OR AB palliat* OR KW palliat*) OR (((TI terminal* OR AB terminal* OR KW terminal*) OR (TI catastroph* OR AB catastroph* OR KW catastroph*) OR (TI death OR AB death OR KW death)) N1 ((TI disease* OR AB disease*) OR (TI care OR AB care) OR (TI ill OR AB ill) OR (TI illness OR AB illness))) OR (TI hospice OR AB hospice) OR (TI hospices OR AB hospices) OR ((TI end OR AB end) N1 (TI life OR AB life)) OR (TI end-of-life OR AB end-of-life) OR (TI "medical complexit*" OR AB "medical complexit*" OR KW "medical complexit*") OR (TI "complex medical condition*" OR AB "complex medical condition*" OR KW "complex medical condition*") OR (TI "complex chronic condition*" OR AB "complex chronic condition*" OR KW "complex chronic condition*") OR (TI "multiple complex chronic" OR AB "multiple complex chronic" OR KW "multiple complex chronic") OR (TI "medical fragility" OR AB "medical fragility" OR KW "medical fragility") OR (TI life-threatening OR AB life-threatening OR KW life-threatening) OR (TI life-limiting OR AB life-limiting OR KW life-limiting) OR (TI "long term hospitalization" OR AB "long term hospitalization" OR KW "long term hospitalization") OR (TI dying OR AB dying OR KW dying)) | 45,813 |
| S24 | ((TI transplantation OR AB transplantation OR KW transplantation) OR (TI "end-stage organ failure" OR AB "end-stage organ failure" OR KW "end-stage organ failure") OR (TI "cystic fibrosis" OR AB "cystic fibrosis" OR KW "cystic fibrosis") OR (TI "Duchenne Muscular Dystrophy" OR AB "Duchenne Muscular Dystrophy" OR KW "Duchenne Muscular Dystrophy") OR (TI "Epidermolysis Bullosa" OR AB "Epidermolysis Bullosa" OR KW "Epidermolysis Bullosa") OR (TI "Neuromuscular Disorder*" OR AB "Neuromuscular Disorder*" OR KW "Neuromuscular Disorder*") OR (TI "Neurodegenerative Disorder*" OR AB "Neurodegenerative Disorder*" OR KW "Neurodegenerative Disorder*") OR (TI "Progressive Metabolic Disorder*" OR AB "Progressive Metabolic Disorder*" OR KW "Progressive Metabolic Disorder*") OR (TI "Advanced Cancer" OR AB "Advanced Cancer" OR KW "Advanced Cancer") OR (TI "invasive cancer" OR AB "invasive cancer" OR KW "invasive cancer") OR (TI Metastas* OR AB Metastas* OR KW Metastas*) OR (((TI child* OR AB child* OR KW child*) OR (TI p#ediatric OR AB p#ediatric OR KW p#ediatric)) N1 ((TI leukemia* OR AB leukemia* OR KW leukemia*) OR (TI leukaemia* OR AB leukaemia* OR KW leukaemia*) OR (TI cancer* OR AB cancer* OR KW cancer*) OR (TI malignan* OR AB malignan* OR KW malignan*) OR (TI myeloma OR AB myeloma OR KW myeloma) OR (TI lymphoma OR AB lymphoma OR KW lymphoma) OR (TI meningioma OR AB meningioma OR KW meningioma) OR (TI seriously-ill OR AB seriously-ill OR KW seriously-ill))) OR (TI "Severe Cerebral Palsy" OR AB "Severe Cerebral Palsy" OR KW "Severe Cerebral Palsy") OR (TI "Multimorphic Syndromes" OR AB "Multimorphic Syndromes" OR KW "Multimorphic Syndromes") | 26,707 |
| S25 | (MA Bereavement) | 2,686 |
| S26 | ((TI bereaved OR AB bereaved) OR (TI bereavement OR AB bereavement ) OR (TI grief OR AB grief) OR (TI grieving OR AB grieving) OR (TI mourning OR AB mourning) OR (TI cancer-bereave* OR AB cancer-bereave*) OR (TI Pre-loss OR AB Pre-loss) OR (TI deathbed OR AB deathbed) OR (TI death-bed OR AB death-bed)) | 26,875 |
| S27 | (((TI recent OR AB recent OR KW recent) OR (TI before OR AB before OR KW before) OR (TI after OR AB after OR KW after) OR (TI traumati* OR AB traumati* OR KW traumati*) OR (TI child* OR AB child* OR KW child*) OR (TI cancer OR AB cancer OR KW cancer) OR (TI loved OR AB loved OR KW loved)) N0 ((TI loss OR AB loss OR KW loss) OR (TI death OR AB death OR KW death))) | 8,234 |
| S28 | S22 OR S23 OR S24 OR S25 OR S26 OR S27 | 98,766 |
| S29 | S17 AND S18 AND S21 AND S28 | 3,216 |
| S30 | (MA “Randomized Clinical Trials”) | 68,8 |
| S31 | (MJ “Clinical Trials”) | 8,018 |
| S32 | TI clinic* N1 trial* OR AB clinic* N1 trial* | 42,79 |
| S33 | TI randomi* control* trial* OR AB randomi* control* trial* | 57,544 |
| S34 | TI allocat* random* OR AB allocat* random* OR DE allocat* random* | 5,96 |
| S35 | TI random* allocat* OR AB random* allocat* OR MA random* allocat* | 14,158 |
| S36 | TI placebo OR AB placebo OR MA placebo | 46,132 |
| S37 | S30 OR S31 OR S32 OR S33 OR S34 OR S35 OR S36 | 138,395 |
| S38 | S29 AND S37 | 85 |
| S39 | S29 | 97 |
| S40 | S38 OR S39 | 105 |
| **ACCESS DATE:** 4th July 2024  **DATABASE:** Cochrane Central Register of Controlled Trials (CENTRAL)  **PLATFORM:** Wiley  **SEARCH FILTER:** None | | |
| ID | Search | Hits |
| #1 | [mh ^Psychotherapy/MT] OR [mh ^"Psychotherapy, Brief"] OR [mh ^"Professional-Family Relations"] OR [mh ^"Adaptation, Psychological"] OR [mh ^"Stress, Psychological"/TH] | 10222 |
| #2 | (psychotherap*:ti,ab,kw OR psychoeducat*:ti,ab,kw OR psychooncolog*:ti,ab,kw) | 24016 |
| #3 | ((psychological:ti,ab,kw OR psychosocial:ti,ab,kw OR famil*:ti,ab,kw OR "stress management":ti,ab,kw) NEXT (therap*:ti,ab,kw OR treatment*:ti,ab,kw OR intervention*:ti,ab,kw)) | 14468 |
| #4 | [mh ^"Mental Health"] | 3305 |
| #5 | [mh ^"Cognitive Behavioral Therapy"] OR [mh ^"Behavior Therapy"/TH] | 11847 |
| #6 | ((cognitive*:ti,ab,kw OR "cognitive behavioral":ti,ab,kw OR cognitive-existential:ti,ab,kw OR existential:ti,ab,kw OR dignity:ti,ab,kw) NEXT therap*:ti,ab,kw) | 11169 |
| #7 | [mh ^"Acceptance and Commitment Therapy"] | 478 |
| #8 | ((dignity:ti,ab,kw OR interpersonal:ti,ab,kw OR "acceptance and Commitment":ti,ab,kw) NEXT therap*:ti,ab,kw) | 2225 |
| #9 | [mh ^"Relaxation Therapy"] | 1652 |
| #10 | ("life review":ti,ab,kw OR "meaning making":ti,ab,kw OR "emotional regulation":ti,ab,kw OR mindfulness:ti,ab,kw OR coping:ti,ab,kw OR conversation*:ti,ab,kw OR "group discussion":ti,ab,kw) | 27774 |
| #11 | [mh ^"Problem Solving"] | 1830 |
| #12 | ("problem solving":ti,ab,kw OR problem-solving:ti,ab,kw) | 7032 |
| #13 | [mh ^"Social Support"] | 4250 |
| #14 | ("support*" NEXT (intervention* OR therap*)):ti,ab,kw | 2034 |
| #15 | [mh ^"Advance Care Planning"] | 362 |
| #16 | Advance Care Planning:ti,ab,kw | 1192 |
| #17 | #1 OR #2 OR #3 OR #4 OR #5 OR #6 OR #7 OR #8 OR #9 OR #10 OR #11 OR #12 OR #13 OR #14 OR #15 OR #16 | 83005 |
| #18 | [mh infants] OR [mh ^"Infant, Newborn"] OR [mh Child] OR [mh ^"Child, Preschool"] OR [mh ^Pediatrics] OR [mh adolescent] OR [mh "congenital, hereditary, and neonatal diseases and abnormalities"] OR [mh ^minors] | 223124 |
| #19 | (infant*:ti,ab,kw OR infants:ti,ab,kw OR infancy:ti,ab,kw OR newborn*:ti,ab,kw OR newborns:ti,ab,kw OR new-born*:ti,ab,kw OR perinat*:ti,ab,kw OR neonat*:ti,ab,kw OR baby*:ti,ab,kw OR babies:ti,ab,kw OR toddler*:ti,ab,kw OR toddlers:ti,ab,kw OR boys:ti,ab,kw OR boyhood:ti,ab,kw OR kids:ti,ab,kw OR child:ti,ab,kw OR child-related:ti,ab,kw OR childhood*:ti,ab,kw OR children*:ti,ab,kw OR adolescent*:ti,ab,kw OR adolescents:ti,ab,kw OR adolescence*:ti,ab,kw OR juvenile*:ti,ab,kw OR youth*:ti,ab,kw OR teen:ti,ab,kw OR teens*:ti,ab,kw OR pubescent*:ti,ab,kw OR pubescence:ti,ab,kw OR pediatric:ti,ab,kw OR pediatrics:ti,ab,kw OR paediatric:ti,ab,kw OR paediatrics:ti,ab,kw OR peadiatric:ti,ab,kw OR peadiatrics:ti,ab,kw) | 373314 |
| #20 | #18 OR #19 | 386095 |
| #21 | [mh ^Parents/PX] OR [mh ^Parenting] OR [mh ^"Parent-Child Relations"] OR [mh ^fathers/PX] OR [mh ^mothers/PX] OR [mh ^"Single parent"/PX] OR [mh ^Family] OR [mh ^"Family Therapy"] OR [mh ^"Legal Guardians"] OR [mh ^Caregivers/PX] | 10206 |
| #22 | (parent:ti,ab,kw OR parents:ti,ab,kw OR mother*:ti,ab,kw OR mothers:ti,ab,kw OR father*:ti,ab,kw OR fathers:ti,ab,kw OR families*:ti,ab,kw OR caregiver*:ti,ab,kw OR caregivers:ti,ab,kw OR care-giver:ti,ab,kw OR care-givers:ti,ab,kw OR carers*:ti,ab,kw OR carer*:ti,ab,kw OR caring:ti,ab,kw OR caregiving:ti,ab,kw OR relatives*:ti,ab,kw OR ("legal" NEXT guardia*):ti,ab,kw) | 94953 |
| #23 | #21 OR #22 | 95970 |
| #24 | [mh ^"Palliative Care"/PX] OR [mh ^"Hospice Care"/PX] OR [mh ^"Precursor Cell Lymphoblastic Leukemia-Lymphoma"/PX] OR [mh ^Death] OR [mh ^Leukemia/PX] OR [mh ^Neoplasms/PX] OR [mh ^"Stem Cell Transplantation"/PX] OR [mh ^"Hematopoietic Stem Cell Transplantation"/PX] OR [mh ^"Terminally Ill"] | 2095 |
| #25 | (palliat*:ti,ab,kw OR ((terminal*:ti,ab,kw OR catastroph*:ti,ab,kw OR death:ti,ab,kw) NEXT (disease*:ti,ab,kw OR care:ti,ab,kw OR ill:ti,ab,kw OR illness:ti,ab,kw)) OR hospice:ti,ab,kw OR hospices:ti,ab,kw OR (end:ti,ab,kw NEAR/2 life:ti,ab,kw) OR end-of-life:ti,ab,kw OR ("medical" NEXT complexit*):ti,ab,kw OR ("complex medical" NEXT condition*):ti,ab,kw OR ("complex chronic" NEXT condition*):ti,ab,kw OR "multiple complex chronic":ti,ab,kw OR "medical fragility":ti,ab,kw OR life-threatening:ti,ab,kw OR life-limiting:ti,ab,kw OR "long term hospitalization":ti,ab,kw OR dying:ti,ab,kw) | 21526 |
| #26 | [mh ^Transplantation/PX] OR [mh ^"End Stage Liver Disease"/PX] OR [mh ^"Cystic Fibrosis"/PX] OR [mh ^"Muscular Dystrophy, Duchenne"/PX] OR [mh ^"Epidermolysis Bullosa"/PX] OR [mh ^"Neuromuscular Diseases"/PX] OR [mh ^"Neurodegenerative Diseases"/PX] OR [mh ^"Cerebral Palsy"/PX] | 160 |
| #27 | (transplantation:ti,ab,kw OR "end-stage organ failure":ti,ab,kw OR "cystic fibrosis":ti,ab,kw OR "Duchenne Muscular Dystrophy":ti,ab,kw OR "Epidermolysis Bullosa":ti,ab,kw OR ("Neuromuscular" NEXT Disorder*):ti,ab,kw OR ("Neurodegenerative" NEXT Disorder*):ti,ab,kw OR ("Progressive Metabolic" NEXT Disorder*):ti,ab,kw OR "Advanced Cancer":ti,ab,kw OR "invasive cancer":ti,ab,kw OR Metastas*:ti,ab,kw OR ((child*:ti,ab,kw OR p?ediatric:ti,ab,kw) NEAR/2 (leukemia*:ti,ab,kw OR leukaemia*:ti,ab,kw OR cancer*:ti,ab,kw OR malignan*:ti,ab,kw OR myeloma:ti,ab,kw OR lymphoma:ti,ab,kw OR meningioma:ti,ab,kw OR seriously-ill:ti,ab,kw)) OR "Severe Cerebral Palsy":ti,ab,kw OR "Multimorphic Syndromes":ti,ab,kw) | 95578 |
| #28 | [mh ^Bereavement] | 193 |
| #29 | (bereaved:ti,ab,kw OR bereavement:ti,ab,kw OR grief:ti,ab,kw OR grieving:ti,ab,kw OR mourning:ti,ab,kw OR cancer-bereave*:ti,ab,kw OR Pre-loss:ti,ab,kw OR deathbed:ti,ab,kw OR death-bed:ti,ab,kw) | 1249 |
| #30 | ((recent:ti,ab,kw OR before:ti,ab,kw OR after:ti,ab,kw OR traumati*:ti,ab,kw OR child*:ti,ab,kw OR cancer:ti,ab,kw OR loved:ti,ab,kw) NEXT (loss:ti,ab,kw OR death:ti,ab,kw)) | 1551 |
| #31 | #24 OR #25 OR #26 OR #27 OR #28 OR #29 OR #30 | 115914 |
| #32 | #17 AND #20 AND #23 AND #31 | 606 |
| #33 | #17 AND #20 AND #23 AND #31 en Ensayos | 587 |
